# Quantitative assessment of porin-mediated solute transport in biomimetic membranes

**DOI:** 10.1007/s00253-025-13666-0

**Published:** 2025-12-19

**Authors:** Maike Scherer, Teena Tom Dieck, Leila Pourtalebi Jahromi, Robert Schober, Maximilian Schäfer, Kathrin Castiglione

**Affiliations:** 1 Institute of Bioprocess Engineering, Friedrich-Alexander-Universität Erlangen-Nürnberg, Paul-Gordan-Straße 3, Erlangen, 91052 Germany; 2 Institute for Digital Communications, Friedrich-Alexander-Universität Erlangen-Nürnberg, Erlangen, Germany; 3 Chair of Pharmaceutical Biology, Department of Biology, Friedrich-Alexander-Universität Erlangen-Nürnberg, Erlangen, Germany

**Keywords:** Porins, Polymersomes, Mass transport, Luminescence, *Gaussia* luciferase, Nanoreactor

## Abstract

**Abstract:**

Porins govern nutrient uptake and antibiotic influx in Gram-negative bacteria, making their characterization critical for understanding permeability, resistance mechanisms, and structure-function relationships. From a biotechnological point of view, they are effective tools for modulating the transport of substances across the outer bacterial membrane or for building catalytically active nanoreactors and biosensors. Quantitative data on mass transport through membranes is of great interest, but not trivial to obtain, as in vivo analyses are confounded by cellular complexity and variability. Here, we present a synthetic bottom-up approach, based on polymersomes containing reconstituted purified porins, enabling direct, quantitative measurement of substrate translocation, while minimizing interferences from native processes. Encapsulation of *Gaussia* luciferase allowed real-time monitoring of coelenterazine (CLZ) translocation across the polymeric membrane in the absence and presence of porins. The typically flash-type luciferase kinetics adapts a glow-type light emission profile, whose signal increases over time. This allows conclusions to be drawn about the substrate concentration accessible to the enzyme, enabling quantitative calculations of the transport rates. The novel approach was exemplarily used to compare the transport characteristics of three *Escherichia coli* porins: Outer membrane protein F (OmpF), a deletion variant selected for larger pore size OmpF∆, and Phosphoporin E (PhoE). OmpF∆ exhibited the highest transport rate of 78 molecules s^−1^ per porin trimer, exceeding OmpF (10.8 molecules s^−1^) more than sevenfold, whereas PhoE showed a lower rate of 2.8 molecules s^−1^ for the neutral CLZ substrate. Analysis of two CLZ derivatives of slightly higher molecular mass and notably greater hydrophobicity revealed that transport through OmpF and OmpF∆ was reduced by half, whereas PhoE exhibited lower selectivity for the selected substrates.

**Graphical Abstract:**

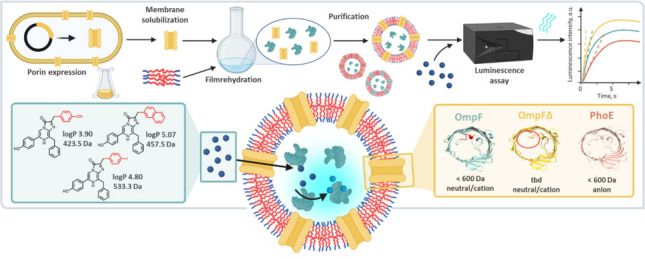

**Key points:**

• *Synthetic polymersomes enable direct, quantitative analysis of porin transport*

• *OmpF∆ exhibits a sevenfold higher molecular flux than wildtype OmpF porins*

• *The assay provides a versatile platform to study porin selectivity and permeability*

**Supplementary information:**

The online version contains supplementary material available at 10.1007/s00253-025-13666-0.

## Introduction

The bacterial cell envelope is a multilayered structure separating the cytoplasm of cells from extracellular compounds, ensuring mechanical integrity, protection, and viability (Hancock 1997; Zgurskaya et al. 2015). Consequently, it functions as a permeability barrier between the interior of cells and their surrounding environment yet permits the passage of small molecules. While the envelope of Gram-positive bacteria, like *Bacillus* species, consists of a cytoplasmic membrane and a thick peptidoglycan layer, Gram-negative species, such as *Escherichia coli* (*E. coli*), have an outer and an inner membrane with a thin peptidoglycan layer in between (Sun et al. 2022; Bos et al. 2007; Hancock and Bell 1988). The outer membrane has a characteristic architecture, consisting of a phospholipid bilayer whose outer leaflet is decorated with lipopolysaccharides. It is also endowed with two distinct classes of proteins: lipoproteins reaching into the compartment between the inner and the outer membrane (periplasm) and outer membrane proteins (Omps) spanning the phospholipid bilayer (Sun et al. 2022). Indeed, atomic force microscopy revealed that Omps cover approximately 70% of the cell surface (Jarosławski et al. 2009). Omps mediate both passive and active transport of small molecules, enabling the selective uptake and expulsion of solutes (Delcour 2009). Among these, porins represent a specialized subset of Omps that facilitate strictly passive diffusion, distinguishing themselves by their selective yet energy-independent transport mechanisms. They serve as key gateways for nutrient uptake and antibiotic influx (Nakae 1986), making them crucial in both physiology and pharmacology, as recently reviewed (Nakae 1986; Sun et al. 2022; Lithgow et al. 2023; Ghai 2024, 2023).

**Fig. 1:**
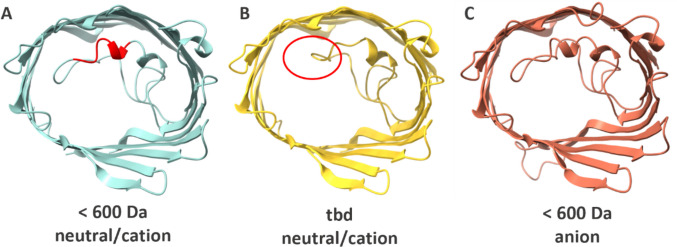
AlphaFold (Abramson et al. 2024) predicted structures of OmpF (**A**) (PDB ID: 2OMF), OmpF∆ (**B**) (PDB ID: 1GFN), and PhoE (**C**) (PDB ID: 1PHO), visualized with ChimeraX. The amino acids 109–114 deleted in OmpF∆ are highlighted in red in (**A**). The size exclusion limits and selectivity of the porins are indicated below the structures. The respective value of the OmpF∆ variant is to be determined (tbd). In order to highlight the eyelet region of the porins, background loops are hidden in the visualization

Porins have been found to be highly conserved across diverse species reflecting their essential role in cell survival and adaptation (Sun et al. 2022; Hancock 1987). The dominant type of porins are general porins like the outer membrane protein F (OmpF) (Fig. 1A) and phosphoporin E (PhoE) (Fig. 1C) from *E. coli*, which are closely related and share 61% sequence identity (Mizuno et al. 1983). OmpF is one of the most extensively researched porins and serves as a model system for understanding passive diffusion of small molecules across bacterial membranes. Porins form water-filled channels that facilitate the passive diffusion of small hydrophilic molecules across the outer membrane, down their concentration gradient (Nikaido 2003; Choi and Lee 2019), while their selectivity is influenced by pore size, charge distribution, and gating behavior (Pagès et al. 2008). General porins typically have a trimeric structure that is defined by 16-stranded ß-barrels forming three channels (Schulz 2002; Garavito and Rosenbusch 1980). Responsible for the constriction of the pore is the loop L3, shown for three porins in Fig. 1, which extends into the interior of the pore and establishes an eyelet at half the height of the porin, which results in an hourglass-shaped passage. This loop determines both the size exclusion limit and the charge-based selectivity of the porin, since negatively charged residues on the loop interact with positive charges on the opposite barrel wall thereby creating a defined opening with an asymmetric electrostatic environment (Eppens et al. 1997; Cowan et al. 1992; Delcour 2009; Phale et al. 2001). While OmpF is cation-selective, PhoE pores are anion-selective (Benz et al. 1985; Jap 1989). The structural knowledge of porins is well established, but quantitative functional transport analysis in a biomimetic, controlled environment remains limited. Understanding how pore size and selectivity affect solute permeability can inform antibiotic uptake and resistance (James et al. 2009; Prajapati et al. 2021), drug design and pharmacokinetics (Gong et al. 2019), biotechnological or synthetic biological applications (Klermund et al. 2017), biosensing (Howorka and Siwy 2009), and transport modelling (Chowdhury et al. 2018).

Being key players in studies of antibiotic uptake and resistance, targeted mutations of porins offer a powerful strategy to dissect structure-function relationships and expand the current repertoire of characterized porins. One such example is OmpF∆, a variant featuring a five amino acid (AA) deletion at the constriction loop L3 (Fig. 1B), which results in an increased pore diameter and improved solute permeability (Saint et al. 1996). While this variant highlights the potential of rational porin engineering, the absolute mass transport characteristics of OmpF∆ remain to be quantitatively resolved – underscoring the need for experimental platforms that can accurately assess the functional impact of porin modifications. However, realizing such functional studies requires reliable recombinant expression of structurally intact porins – an endeavor that remains technically challenging due to their inherent hydrophobicity and potential cellular toxicity on the host organism (Dilworth et al. 2018; Overbeeke and Lugtenberg 1980; Link and Georgiou 2007). Efficient overexpression often requires overcoming bottlenecks in membrane insertion and solubility, necessitating the use of tightly regulated expression systems and tailored purification strategies (Errey and Fiez-Vandal 2020).

The analysis of mass transport through membrane porins is a complex process, which faces many challenges. These include the need to consider complex and substrate-dependent selectivity, sensitivity to environmental conditions, and the difficulty of isolating and measuring their function. The analysis of membrane porins in whole-cell permeability assays poses significant complexities, as porin activity frequently intersects with other transport phenomena. The inherent heterogeneity of native membranes further complicates direct measurement, necessitating sophisticated analytical techniques (Casas-Rodrigo et al. 2025; Prajapati et al. 2021; Winterhalter and Ceccarelli 2015). To gain an understanding of the underlying mechanism of mass transport, bottom-up synthetic biology approaches reduce the number of parameters involved. The reconstitution into artificial membranes, e.g., made from lipids or polymers, allows to determine the mass transport of isolated porins. Methods such as single-channel conductance measurements (Saint et al. 1996; Cama et al. 2015), liposome swelling (Phale et al. 2001; Pagès et al. 2008), or planar lipid bilayer measurements (also known as black lipid membrane (BLM) technique) (Bajaj et al. 2017; Gutsmann et al. 2015) gave insight into the size exclusion limit of OmpF to approximately 600 Da (Nikaido and Rosenberg 1983). However, conductance measurements are not inherently proportional to absolute solute flux, as ionic currents capture channel electrophysiology rather than diffusional transport kinetics of bulky substrates (Delcour 2009). While liposome swelling allows for a semi-quantitative and indirect detection, this approach is often complicated by leakage due to the high fluidity and permeability of the liposomal membrane, which can lead to passive solute diffusion and reduced assay sensitivity. In contrast, BLM measurements provide a direct and quantitative detection method of single-channel conductance and selectivity ratios (Girard-Egrot and Maniti 2021). Nevertheless, its low throughput, technical complexity, and limitation to ion-conducting channels render it suboptimal for broader mass transport studies. Moreover, values from electrophysiological measurements do not necessarily correlate with transport properties, and each assay provides only an apparent pore size, specific to the compound for which permeability is measured (Eppens et al. 1997).

A suitable alternative to liposomes are vesicles made from synthetic amphiphilic block copolymers, so-called polymersomes (Discher and Eisenberg 2002). Like liposomes, which are composed of natural or synthetic phospholipids, polymersomes spontaneously self-assemble in aqueous solutions (Akbarzadeh et al. 2013; Discher and Eisenberg 2002). Independent of their building blocks, both vesicle types are able to encapsulate both hydrophilic and hydrophobic cargos within their lumen and membrane, respectively (Shen et al. 2013; Rideau et al. 2018). Nevertheless, polymersomes offer several key advantages compared to liposomes. While liposomes are described as problematically leaky due to their high lateral fluidity linked to the low molecular mass of the polymeric monomers and thin membranes (3–5 nm) (Le Meins et al. 2011), polymersomes offer increased stability and lower basic permeability due to their usually much thicker membrane (3–40 nm) (Palivan et al. 2016; Poschenrieder et al. 2017). These properties render polymersomes ideal candidates to provide a robust, tuneable platform to reconstitute and study membrane proteins (Klermund et al. 2017; Meyer et al. 2021; Lo and Zeng 2023). In previous studies, OmpF has been repeatedly successfully reconstituted with full functionality into poly(2-methyloxazoline)_15_-poly(dimethylsiloxane)_68_-poly(2-methyloxazoline)_15_ (PMOXA-PDMS-PMOXA) membranes to generate nanoreactors for synthetic applications (Nardin et al. 2000; Klermund et al. 2017; Grzelakowski et al. 2009; Itel et al. 2015; Klermund and Castiglione 2018; Meyer et al. 2021). The compartmentalization of inhibited reactions in multi-enzyme reaction cascades has been demonstrated to enhance the yield of products such as CMP-N-acetylneuraminic acid (Klermund et al. 2017). To facilitate the tailored translocation of substrates and products into and out of nanoreactors, it is indispensable to expand the portfolio of membrane porins in terms of size and selectivity.

Building on this concept, we designed a bioluminescence-based assay in which *Gaussia* luciferase (GLuc) is encapsulated within polymersomes, serving as a sensitive reporter enzyme to monitor substrate translocation through membrane porins. GLuc is a small (~19.9 kDa), naturally secreted luciferase derived from the marine copepod *Gaussia princeps* (Tannous et al. 2005). It exhibits exceptionally high bioluminescent activity, producing intense light emission upon irreversible oxidation of its substrate coelenterazine (CLZ) only dependent on oxygen as cosubstrate (Inouye and Sahara 2008; Tannous 2009; Dijkema et al. 2021). Due to its high sensitivity and low background signal, GLuc is widely used as a robust and non-invasive reporter protein (Tannous 2009), which permeates through membranes easily. Even though GLuc showed a narrow substrate specificity (Inouye and Sahara 2008; Inouye et al. 2013), the developed assay allows the study of different substrate derivatives of differing size and properties.

In this work, we used the GLuc-based assay to exemplarily compare the transport characteristics of three porins with different selectivity and size exclusion limits: OmpF, OmpF∆, and PhoE. The recombinantly expressed and purified porins were reconstituted into polymersomes, followed by analysis of the insertion of the different porins and their mass transport properties. To enable quantitative analysis, we propose and validate a transport model tailored to this system. To this end, we take advantage of the fact that the thick polymer membrane imposes a strong mass transport limitation, which converts the flash kinetics of the luciferase into a glow-type enzyme response (Meyer et al. 2021). Next to the standard substrate CLZ, we present results using uncharged CLZ derivatives of different sizes and hydrophobicity to probe transport behavior.

## Methods

### Chemicals and reagents

Chemicals, as well as the substrate CLZ, were purchased from Carl Roth (Karlsruhe, Germany). The polymer (PMOXA_15_-PDMS_68_-PMOXA_15_) was obtained from Polymer Source Inc. (Dorval, Canada). Detergents were purchased from Bachem AG (Bubenhof, Schweiz). The substrate derivatives were purchased from Biotium (Fremont, USA).

### Plasmids and bacterial strains

The vector suitable for bacterial expression of *Gaussia* luciferase (GLuc) pET28a-his-Gluc-iLID was a gift from Avi Schroeder (Addgene plasmid #172,096; http://n2t.net/addgene:172096; RRID:Addgene_172096) (Adir et al. 2022) (Watertown, MA, USA), including the GLuc mutations M43L, M110L, and lacking the secretion signal peptide sequence, amino acids 2–17. The GLuc variant exhibits a brighter and longer half-life of illumination, glow-like kinetics, and improved expression yields (Welsh et al. 2009). Since the plasmid encodes for a fusion protein of GLuc and iLID, we introduced a stop codon in between the two proteins at position 175 via Q5® site-directed mutagenesis (New England Biolabs 2015) to ensure expression of only GLuc (result not shown). For GLuc expression, the strain *E. coli* BL21 (DE3) SHuffle® T7 Express (New England Biolabs, Frankfurt am Main, Germany) was employed.

The porins OmpF, OmpF∆, and PhoE were expressed in *E. coli* BL21 (DE3) omp8 (Prilipov et al. 1998) as a fusion of the PhoE leader peptide and a His_6_-tag following the leader peptidase cleavage site on a pET21-Plasmid (Schwarzer et al. 2018). The strain was kindly provided by Prof. Ulrich Schwaneberg (Institute of Biotechnology, RWTH Aachen, Germany).

### Cell cultivation, protein purification and analysis

#### The catalyst *Gaussia* luciferase

The pre-culture was performed in 20 mL lysogeny broth (LB) medium supplemented with 50 mg L^−1^ kanamycin (overnight cultivation at 37 °C and 180 rpm in an incubation shaker with 50 mm orbit diameter (Multitron, Infors HT, Bottmingen, Switzerland)). The main culture was inoculated in fresh LB medium supplemented with 50 mg L^−1^ kanamycin to an optical density at 600 nm (OD_600_) of 0.1 and cultivated at 30 °C, 155 rpm at 50 mm orbit diameter (Multitron, Infors HT, Bottmingen, Switzerland) until an OD_600_ of 0.8. The protein synthesis was induced using 0.5 mM isopropyl‐β‐D‐thiogalactopyranoside (IPTG) and performed at 18 °C for 20 h at 155 rpm.

After protein expression, the cells were harvested at 4500 × g for 15 min and resuspended in 5 mL binding buffer (Tris-HCl 50 mM, pH 7.6, imidazole 20 mM) per g cell wet weight. The cells were lysed by sonication with a Sonoplus MS73 (Bandelin, Berlin, Germany) for 2 × 5 min, 1 s cycles, 90% amplitude on ice and cleared by centrifugation at 15,557 × g for 45 min at 4 °C. The supernatant including GLuc was purified via immobilized metal affinity chromatography (IMAC) using binding buffer (Tris-HCl 50 mM, pH 7.6, imidazole 20 mM) and elution buffer (Tris-HCl 50 mM, pH 7.6, imidazole 100 mM). The buffer was changed against storage buffer (50 mM Tris-HCl, pH 7.6, 300 mM NaCl, 10 mM ethylendiamintetraessigsäure (EDTA)) upon purification using PD-10 desalting columns (Cytiva, Marlborough, USA).

#### Porins

All of the porin variants were expressed and purified as described by Golombek and Castiglione (Golombek and Castiglione 2020) as a fusion of the PhoE leader peptide and a His_6_-tag following the leader peptidase cleavage site. In brief, purification was achieved via membrane solubilization with 3% (w/v) N,N-dimethyldodecylamine N-oxide (LDAO), followed by several ultracentrifugation steps and subsequent IMAC purification. After purification, the solubilized porins were stored in a tris-based buffer (Tris-HCl 20 mM, pH 8.0, NaCl 50 mM, 0.4% (v/v) n-Octyl-oligo-oxyethylene (O-POE)).

#### Bis-Tris polyacrylamide gel electrophoresis

For the analysis of protein expression, discontinuous Bis-Tris gels were prepared as described by Fritzsche et al. (2024). The only difference was that the denaturation of the porin samples was performed at a higher temperature of 99 °C for 15 min, ensuring complete denaturation of the sample (Schwarzer et al. 2018). GLuc samples were denatured at 95 °C for 5 min. As a standard, the Precision Plus Dual Color Standard (BIORAD, Hercules, USA) was used.

#### Determination of protein concentration

The protein concentration of GLuc and the porins was determined using a NanoDrop-1000 (Thermo Fisher, Waltham, USA) and corrected using densitometric analysis of the generated PAGE gels.

### Formation and analysis of polymersomes

In this work, polymersomes in four different compositions were analyzed, each of them encapsulating GLuc. Of these, three contained either reconstituted OmpF, OmpF∆, or PhoE, while the control polymersomes contained no porin in the polymeric membrane. In the following, these configurations will be referred to as PolGLOmpF, PolGLOmpF∆, PolGLPhoE, and PolGLControl, respectively.

#### Film rehydration

The polymersomes were fabricated via film rehydration. Before polymersome formation, a stock solution of 5.13 µM of the designated porins with buffer (50 mM NaCl, pH 8.0, 20 mM Tris-HCl, 0.6% (v/v) O-POE) was prepared. Ten milliliters of a 1% (w/v) PMOXA_15_-PDMS_68_-PMOXA_15_ solution in ethanol was transferred into a 100-mL round-bottom flask and dried in a rotary evaporator (Laborata 4002, Heidolph, Schwabach, Germany) at 130 mbar and 75 rpm at 40 °C. The resulting thin polymer film was rehydrated with a solution of 10 mL consisting of 9.8 mL of a 0.1 mg mL^−1^ GLuc solution in phosphate buffered saline (PBS) solution and 0.2 mL of the respective porin stock solution. To produce control polymersomes (PolGLControl), O-POE was added to match the final O-POE concentration of 0.012% (v/v) in the solution when adding the porin stock solution. The resulting mixture was stirred overnight at 4 °C and then extruded 2 times through a polycarbonate (PC) membrane with a 200 nm diameter pore size (Merck, Darmstadt, Germany) using a filter holder for syringes (Carl Roth, Karlsruhe, Germany). Unencapsulated proteins were removed by size-exclusion chromatography (SEC) using a 25 × 200 mm column (Kronlab, Dinslaken) with a Sepharose 4B (GE Healthcare) matrix of 200 mL bed volume and PBS as the mobile phase.

#### Dynamic light scattering

The size and the polydispersity of the polymersome dispersion were determined by dynamic light scattering (DLS) using a ZetaSizer Nano-S (Malvern Instruments Inc., Malvern, UK) at an angle of 173° with 500 µL of a 0.1% (w/v) polymersome dispersion in PBS. A poly dispersity index (PDI) below 0.2 was aimed for to meet the necessary quality criteria. The resulting z-average was used to determine the diameter of the polymersomes.

#### Analysis of porin insertion and GLuc encapsulation via gel electrophoresis

The insertion of porins into the polymeric membrane and the encapsulation of GLuc was analyzed by 12% sodium dodecyl sulfate–polyacrylamide gel electrophoresis (SDS-PAGE). Initially, the polymersomes were concentrated via ultracentrifugation at 125,000 × g and 4 °C for 1 h. The subsequent sample preparation was identical to that in the “Bis-Tris polyacrylamide gel electrophoresis” section, except that Laemmli buffer was used as sample buffer (Laemmli 1970). As standard, the Perfect Protein™ marker (Merck, Darmstadt, Germany) was used. The gel was dyed according to the manufacturer’s instructions using SYPRO™ ruby fluorescent dye (Invitrogen™, Waltham, USA).

#### Analysis of the polymersome concentration

The number of polymersomes per mL was determined using the ZetaView® Twin (Particle Metrix, Inning am Ammersee, Germany) nanoparticle tracking analyzer. The measurements were done at 25 °C in scatter mode, using a 488 nm laser, and with the following configurations: sensitivity of 80%, shutter of 100, max area of 1000, min area of 10, and min brightness of 30.

### Luminescence assay

Luminescence measurements were carried out in a Tecan Infinite® M Plex microplate spectrometer (Tecan, Männedorf, Switzerland) equipped with a Te-Inject™ (Tecan, Männedorf, Switzerland) in luminescence mode using white CLS3610 96-well plates (Corning, New York, USA). CLZ was diluted in PBS from a 1 mM stock solution to a concentration of 160 µM and incubated in the dark for 30 min. Prior to injection, 50 µL of the polymersomes was added to each well in triplicate measurements and incubated in the dark for 15 min. Then, 50 µL of CLZ was injected well-wise, followed by immediate measurement of the luminescence intensity every 2 s over 10 min with an integration time of 500 ms in arbitrary units (a.u.).

For the measurements using free GLuc, the enzyme was prepared at a concentration of 0.05 µM in PBS. A volume of 50 µL was added to each well in triplicate, followed by a 15 min incubation in the dark. Subsequently, CLZ-substrate was injected in different concentrations of 0.1 µM, 0.5 µM, 1 µM, 2 µM, 3 µM, 5 µM, 7 µM, 10 µM, 15 µM, 20 µM, 25 µM, and 30 µM, followed by immediate measurements of the luminescence intensity every 2 s for 10 s with an integration time of 500 ms.

### Software

Porin structures were modelled using AlphaFold (Abramson et al. 2024) and visualized in UCSF ChimeraX, developed by the Resource for Biocomputing, Visualization, and Informatics at the University of California, San Francisco, with support from the National Institutes of Health R01-GM129325 and the Office of Cyber Infrastructure and Computational Biology, National Institute of Allergy and Infectious Diseases (Meng et al. 2023). The molar mass of the Omps and GLuc, including the His_6_-tag, was calculated using ProtParam (ExPASy, Swiss Institute of Bioinformatics). Densitometric analysis was performed using ImageJ (Schneider et al. 2012) to determine protein purity and insertion of the porins. BioRender was employed for the design of the graphical abstract. GraphPad Prism was used for the figure design and data analysis. SwissADME was employed for the analysis of the P-values of the substrate derivatives (Daina et al. 2017). The chemical structures of CLZ and its derivatives were created using ChemDraw (Revvity Signals).

## Results

### Yields of recombinant porins and GLuc

The results of the recombinantly expressed and purified porins and the enzyme GLuc are summarized in Table 1. The results of the PAGE analyses are displayed in Figs. S1–S4. Protein concentrations of 0.64 mg mL^−1^, 0.20 mg mL^−1^, 2.4 mg mL^−1^, and 0.90 mg mL^−1^ could be determined for OmpF, OmpF∆, PhoE, and GLuc, respectively. The obtained yields of total purified protein per bio wet mass and per liter cell culture for OmpF, OmpF∆, PhoE, and GLuc were 0.11 mg g^−1^ bio wet mass, 0.055 mg g^−1^ bio wet mass, 0.37 mg g^−1^ bio wet mass, and 0.6 mg g^−1^ bio wet mass, or 2.0 mg L^−1^ culture, 0.52 mg L^−1^ culture, and 7.1 mg L^−1^ culture, respectively. Relative to the benchmark porin OmpF, the purification upon expression resulted in a twofold decrease in yield for OmpF∆ and a more than three-fold increase for PhoE.

**Table 1:** Results of the recombinant expression of the porins OmpF, OmpF∆, and PhoE, as well as the enzyme GLuc. Listed are the obtained protein concentrations, the yield per bio wet mass, and the yield per liter cell culture

	C, mg mL^−1^	Yield, mg g^−1^ bio wet mass	Yield, mg L^−1^ culture
OmpF	0.64	0.11	2.0
OmpF∆	0.20	0.055	0.52
PhoE	2.4	0.37	7.1
GLuc	0.88	0.59	4.06

### Analysis of polymersome formation

Following polymersome formation via film rehydration and their subsequent purification, the polymersomes were analyzed regarding their diameter, concentration, and porin reconstitution using DLS, nanoparticle tracking, and densitometry, respectively.

#### Size and concentration

The characterization of the polymersomes regarding their intensity-based *z*-average, the *X* mean, the PDI, and the concentration are listed in Table 2. Following the extrusion of the polymersomes upon film rehydration, SEC was used to remove non-encapsulated protein and to deplete the solution from micelles. Given that the polymeric membrane is approximately 14 nm thick, the diameter of these micelles is estimated to be around 28 nm. Following the SEC, the PDI of the solution was reduced below 0.20 for all the polymersome samples. The size distribution curves before and after SEC obtained from DLS-measurements are displayed in Fig. S5. The results obtained from DLS indicate a successful depletion of micelles from the polymersome solution, since the peak around 28 nm vanishes in all samples. The obtained *z*-averages ranged from 201.7 ± 6.4 nm for PolGLControl to 146.7 ± 0.9 nm for PolGLOmpF. PolGLOmpF∆ and PolGLPhoE resulted in 175.1 ± 3.8 nm and 161.0 ± 3.0 nm, respectively. It was observed that polymersomes with reconstituted porins resulted in a smaller diameter than the control polymersomes PolGLControl. The reliability of these results was further validated by nanoparticle tracking analysis, which yielded *X* mean values that aligned with the z-averages determined by DLS. The concentration of polymersomes was found to be consistent, with all values falling within the range of 10^12^ polymersomes per mL. However, for PolGLPhoE, the concentration employed for the luminescence assay was reduced due to the high deviation of the initial sample. The concentration used for further calculations is included in Table 2.

**Table 2:** Results from size measurements of the polymersome samples PolGLControl, PolGLOmpF, PolGLOmpF∆, and PolGLPhoE. The *z*-average and PDI were obtained from DLS measurements, while the *X* mean and the concentration of the polymersomes per mL resulted from nanoparticle tracking

Sample	*z*-average, nm	*X* mean, nm	PDI	Concentration, pol mL^−1^
PolGLControl	201.7 ± 6.4	172.1 ± 78.6	0.16	3.5*10^12^ ± 4.6*10^11^
PolGLOmpF	146.7 ± 0.9	144.3 ± 73.1	0.10	2.4*10^12^ ± 2.4*10^11^
PolGLOmpF∆	175.1 ± 3.8	175.6 ± 75.1	0.18	1.7*10^12^ ± 2.04*10^11^
PolGLPhoE	161.0 ± 3.0	160.5 ± 65.3	0.15	4.1*10^11^ ± 4.92*10^10^

Following the determination of the diameters of the polymersome samples, the theoretical number of encapsulated enzymes was calculated, assuming statistical encapsulation of the applied enzyme concentration ${c}_{\mathrm{GLuc}}$ of 5.26 µM. First, the total inner volume of one polymersome $P$, ${V}_{\mathrm{P},\mathrm{i}}$, was determined using Eq. 1, where ${d}_{\mathrm{P}}$ denotes the diameter of the polymersome, and ${r}_{\mathrm{M}}$ the thickness of the polymeric membrane of 14 nm:

1 $${V}_{\mathrm{P},\mathrm{i}}= \frac{4}{3}*\pi *{\left(\frac{{d}_{\mathrm{P}}}{2}-{r}_{\mathrm{M}}\right)}^{3}$$

The theoretical number of encapsulated GLuc ${N}_{\mathrm{GLuc},\mathrm{i}}$ was subsequently calculated using Eq. 2 based on the Avogadro number ${N}_{\mathrm{A}}$, the concentration of GLuc ${c}_{\mathrm{GLuc}}$, and the molecular weight ($MW$) of GLuc, which is around 19.9 kDa:

2 $${N}_{\mathrm{GLuc},\mathrm{i}}= \frac{{V}_{\mathrm{P},\mathrm{i}}*{c}_{\mathrm{GLuc}}*{\mathrm{N}}_{\mathrm{A}}}{MW}$$

The theoretically determined number of GLuc enzymes per polymersome ranged from 8.8 ± 0.28 to 2.7 ± 0.017 enzymes per polymersome for PolGLControl and PolGLOmpF. PolGLOmpF∆ and PolGLPhoE resulted in 5.3 ± 0.11 and 4.0 ± 0.073 GLuc enzymes per polymersome, respectively.

#### Insertion into the synthetic membrane

The insertion of the porins was analyzed using densitometry measurements. The PAGE gels obtained from concentrated polymersomes with and without reconstituted porins are displayed in Figs. S6–S9. For all porin-containing polymersome batches PolGLOmpF, PolGLOmpF∆, and PolGLPhoE, porins were visible around 38 kDa.

The densitometric analysis resulted in 0.20 ± 0.021 OmpF-trimers, 0.030 ± 0.004 OmpF∆-trimers, and 0.68 ± 0.082 PhoE-trimers per polymersome, taking the different polymersome concentrations into account. This corresponds to a more than threefold higher insertion of PhoE and a more than sixfold lower insertion of OmpF∆ relative to OmpF. The encapsulation of GLuc could be quantified to 7.3 ± 1.07 GLuc enzymes per polymersome for PolGLControl, which corresponds to the values determined theoretically in the “Size and concentration” section. As a consequence, statistical encapsulation of GLuc inside the polymersomes can be assumed.

In this work, our goal was not to maximize porin density in the polymeric membrane, but to precisely limit incorporation to a maximum of one single porin trimer per polymersome. This deliberate design minimizes substrate accumulation inside the polymersome, thereby preserving the sensitivity and accuracy of the assay. To quantify transport rates using the luminescence assay, the molecular influx must remain below the catalytic capacity of the encapsulated enzymes. This ensures that the enzymatic turnover, rather than substrate saturation, defines the assay’s readout.

### Analysis of the mass transport

**Fig. 2:**
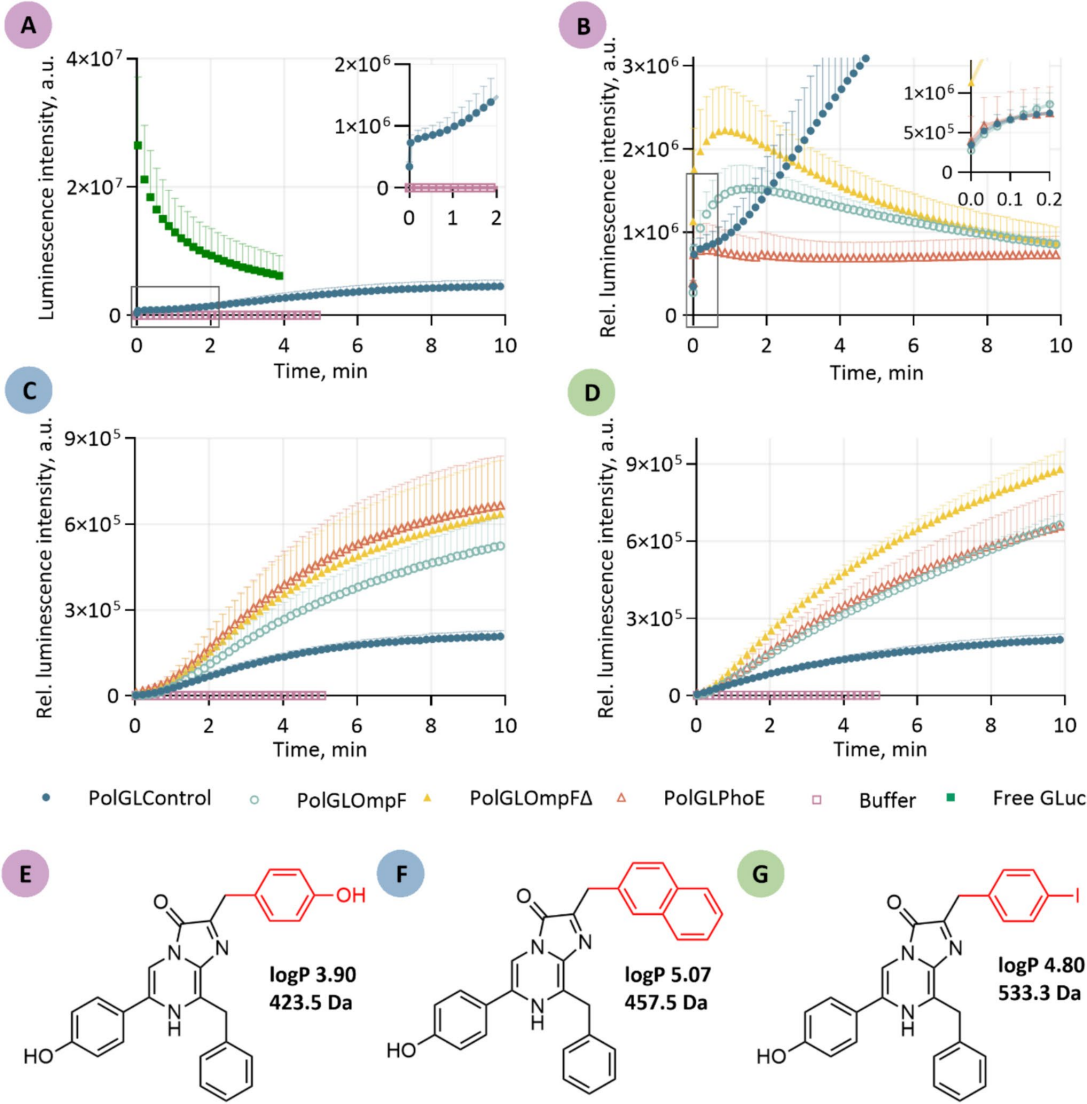
Luminescence intensity over time obtained from the mass transport analysis assay. To facilitate visualization, only every fifth recorded data point is shown in the graphs **A–D,** except for the inset in** B**. Additionally, standard deviations are displayed only above the data points. The flash-type luminescence signal of 0.05 µM free GLuc upon injection of 15 µM coelenterazine (CLZ) is shown in green-filled rectangles in **A**. As a reference, the negative control of polymersomes with no porins PolGLControl (cyan-filled circles) and the buffer control (violet rectangles) are shown in **A** and scaled accordingly. The luminescence intensity relative to the lowest polymersome concentration over time upon the injection of 80 µM CLZ (**B**) to a polymersome solution with reconstituted porins OmpF, OmpF∆, and PhoE resulted in the signal PolGLOmpF (turquoise circles), PolGLOmpF∆ (yellow filled triangles), and PolGLPhoE (red triangles), respectively. The luminescence signal upon mass transport of the CLZ derivative coelenterazine-n (CLZ-n) and coelenterazine-I (CLZ-I) is displayed in **C** and **D**, respectively. The structures, logP values, and molecular masses of the derivatives CLZ, CLZ-n, and CLZ-I are shown in **E**, **F**, and **G**, respectively. We used color coding to allocate the substrates employed in the graphs **A**–**D**: violet for CLZ (**E**), blue for CLZ-n (**F**), and green for CLZ-I (**G**)

To investigate the mass transport of the porins OmpF, OmpF∆, and PhoE reconstituted into polymersomes, a luminescence assay was conducted followed by the derivation of a suitable transport model. The characteristic flash-type kinetics of free GLuc was confirmed in a control experiment for a GLuc concentration of 0.05 µM and a CLZ concentration of 15 µM as illustrated in Fig. 2A (green-filled rectangles), showing a fast declining luminescence signal that is quickly fading. The injection of CLZ into the buffer resulted in a low signal (< 114 a.u., purple rectangles), which is additionally shown in Fig. 2A. Upon encapsulation of GLuc, the enzyme kinetics is shifted towards a longer-lasting light signal, which has been demonstrated in a previous study (Meyer et al. 2021). Since CLZ and CLZ derivatives can permeate through various cell membranes (Shimomura 1997), we examined the control polymersomes PolGLControl to assess the slow and passive background permeability (blue circles) in Fig. 2A and B. Upon an initial rise to a luminescence intensity plateau (highlighted in the inset of Fig. 2A), the signal slowly increases. The initial rise can be attributed to minimal packing defects in the polymeric membrane (Steinkühler et al. 2022), followed by a saturation of hydrophobic CLZ in the polymeric membrane due to its high affinity towards the large hydrophobic PDMS block of the polymer monomers. Once the middle layer of the polymers is saturated, the molecules slowly enter the lumen of the polymersome, resulting in an increase in luminescence intensity.

The bioluminescence intensity signal is influenced by the permeability of the polymeric membrane. When porins are reconstituted, membrane permeability increases, facilitating the diffusional translocation of the substrate. As a result, the light signals display an earlier maximum in luminescence intensity with different slopes, depending on the reconstituted membrane porin (Fig. 2B). Following the peak maximum, the luminescence signal gradually decreases. Figure 2B shows the luminescence intensity relative to the lowest polymersome concentration of PolGLPhoE (Table 2), while the raw data is shown in Figure S11A. For all three reconstituted porins, increased mass transport compared to the control could be observed. Out of the three porins, PolGLPhoE (red triangles) showed the shortest delay with 0.7 min, while the signal reaches its maximum around 0.8 min and 2.6 min for PolGLOmpF∆ (yellow filled triangles) and PolGLOmpF (turquoise circles), respectively. PolGLPhoE and PolGLOmpF experienced similar initial increases (inset Fig. 2B), while PolGLOmpF∆ showed the highest initial increase and overall luminescence intensity. In a biological replicate of PolGLPhoE, the scalability of the assay that has also been shown in the literature (Meyer et al. 2021) resulted in an almost ten times higher luminescence intensity for ten-times more concentrated polymersomes (Figure S11B).

In addition, the mass transport of the two derivatives CLZ-n and CLZ-I was investigated (Fig. 2C and D). These derivatives differ not only in their molecular mass from CLZ (423.5 Da, shown in Fig. 2E), which is 457.5 Da and 533.3 Da for CLZ-n (Fig. 2F) and CLZ-I (Fig. 2G), respectively, but also in their chemical substituents, which influence properties such as polarity, rotational freedom, and membrane affinity. Structural modifications, particularly those affecting hydrophobicity and steric flexibility, have a direct impact on both membrane affinity and interaction with the porins. For instance, CLZ-n, with a logP of 5.07, is significantly more lipophilic than the native CLZ (logP 3.9) and CLZ-I (logP 4.8). LogP represents the decadic logarithm of the partition coefficient (P) of a chemical compound in a two-phase system of n-octanol and water, with more hydrophobic molecules having higher values. Such variations suggest that increased membrane affinity may favor retention within the polymeric membrane, potentially impeding translocation through porins. However, both derivatives remain uncharged, like CLZ. Thus, alterations in mass transport are unlikely to arise from charge selectivity. The structure of CLZ-n is shown in Fig. 2F; the hydroxybenzyl group at the C2 position of the imidazopyrazinone ring of native CLZ is replaced by a ß-naphthyl group. While this modification strengthens membrane interaction, it also introduces greater steric hindrance and reduced conformational flexibility, which may affect the molecule’s ability to traverse the narrow constriction zones of porin channels. For CLZ-I, the hydroxy group of the C2 hydroxybenzyl substituent is substituted by iodine, resulting in the highest molar mass of the three derivatives (Fig. 2G). The luminescence intensity from mass transport measurements of CLZ-n and CLZ-I is depicted in Fig. 2 C and D, respectively. The translocation of the derivatives differs markedly from that of native CLZ (Fig. 2A). Instead of the rapid rise in luminescence intensity reaching a maximum within less than 2 min, the signal increases gradually over the entire 10-min measurement period. However, the facilitated translocation of the derivatives results in the highest slope returned from PolGLPhoE, followed by PolGLOmp∆ and PolGLOmpF, compared to PolGLControl for CLZ. The addition of the derivative CLZ-n resulted in almost identical slopes for PolGLPhoE and PolGLOmpF∆, followed by PolGLOmpF, relative to the control polymersomes. In the case of CLZ-I, PolGLOmpF∆ showed the highest rise in luminescence intensity, followed by PolGLPhoE and PolGLOmpF, exhibiting similar results. PolGLControl shows comparable fluorescence intensities for both CLZ-n and CLZ-I.

### Model for quantification of mass transport

**Fig. 3:**
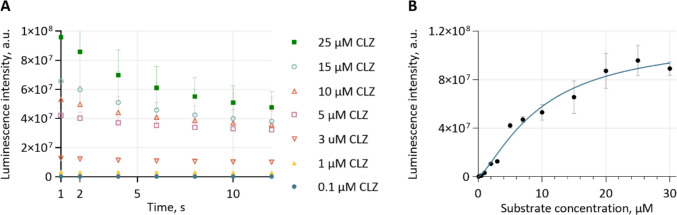
Results of the calibration curve of free GLuc at different substrate concentrations. The luminescence intensity is shown for different coelenterazine (CLZ)-concentrations (**A**). The mean of the extrapolated values of the initial recorded signal are depicted with black dots including the standard deviation of the mean in (**B**). The data was then fitted to a Hill-model, with a r^2^ of 0.979, resulting in a Hill parameter *h* of 1.44 (95% CI [0.83, 2.1]), a v_max_ of 1.14 × 10^8^ (95% CI [8.9 × 10^7^, 2.40 × 10^8^]), and a K_0.5_ of 9.8 µM (95% CI [6.2 µM, 42.9 µM]

To quantify the mass transport of the different porins, the measured luminescence intensity was correlated with the concentration of translocated substrate using a calibration curve generated from free GLuc at varying substrate concentrations ${c}_{\mathrm{S}}$. The complete data of the detected substrate concentrations are shown in Figure S10, while a selection of the raw luminescence results from 0.1 µM, 1 µM, 3 μM, 5 µM, 10 µM, 15 µM, and 25 µM CLZ is included in Fig. 3A. The kinetic of GLuc is characterized by a sharp luminescence peak, occurring almost instantaneously upon substrate addition, that rapidly decays over time (Borum et al. 2025; Rathnayaka et al. 2010). Therefore, the most reliable estimate of an initial rate is the height of the luminescence peak immediately after substrate injection (Dijkema et al. 2021). Measurements were taken 2 s after injection, even though the luminescence peak occurs at approximately 1 s (Harinen et al. 2013). To account for this offset, values were extrapolated by one second to an exponential decay model (Eq. 3) to accurately reflect peak intensity.

3 $$y=a*{exp}^{\left(-{k}_{1}*t\right)}+b$$

The extrapolated calibration curve at 1 s is shown in Table 3 B (black circles).

**Table 3:** Calculated assay sensitivities for the three polymersome batches with reconstituted porins – PolGLOmpF, PolGLOmpF∆, and PolGLPhoE

Polymersomes	Maximal mass transport, s^−1^
PolGLOmpF	49
PolGLOmpF∆	94
PolGLPhoE	68

GLuc is suggested to exhibit kinetic cooperativity (Larionova et al. 2018; Porter and Miller 2012), arising as a result of conformational changes in response to substrate binding (Porter and Miller 2012). Another illustrative example of an enzyme displaying similar behavior is human glucokinase, which possesses a single glucose-binding site yet exhibits a cooperative kinetic response to its substrate (Larion et al. 2012; Whittington et al. 2015). Consequently, we fitted a Hill equation (Eq. 4) to the mean of our calibration curve (blue line in Fig. 3B) of varying substrate concentration ${c}_{\mathrm{S}}$, obtaining an coefficient of determination (${r}^{2}$) of 0.979, a Hill parameter $h$ of 1.4 (95% confidence interval (CI) [0.83, 2.1]) indicating positive cooperativity, a ${v}_{\mathrm{max}}$ of 1.14 × 10^8^ (95% CI [8.9 × 10^7^, 2.4 × 10^8^]), and a ${K}_{0.5}$ of 9.8 µM (95% CI [6.2 µM, 43.1 µM]):

4 $${v}_{\text{Free GLuc}}=\frac{{v}_{\mathrm{max}}*{{c}_{\mathrm{S}}}^{h}}{{{K}_{0.5}}^{h}+{{c}_{\mathrm{S}}}^{h}}$$

In order to adjust the enzyme concentration from the calibration curve to the concentration employed in polymersome experiments, the total amount of enzyme inside the polymersomes ${n}_{\mathrm{GLuc},\mathrm{P}}$ has been determined by the enzyme concentration in the lumen of the polymersomes ${c}_{\mathrm{GLuc},\text{ i}}$ in mg per mL and the total inner volume of the polymersomes ${V}_{\mathrm{total},\text{ i}}$ applied during an experiment (Eq. 5). ${V}_{\mathrm{P},\mathrm{i}}$ denotes the inner volume of one polymersome, ${N}_{\mathrm{P}}$ the concentration of the polymersomes per mL applied, and ${V}_{\mathrm{E}}$ the volume in the well of the experiment:

5 $${n}_{\mathrm{GLuc},\mathrm{P}}={c}_{\mathrm{GLuc},\mathrm{i}}*{V}_{\mathrm{total},\mathrm{i}}={c}_{\mathrm{GLuc},\mathrm{i}}*{V}_{\mathrm{P},\mathrm{i}}*{N}_{\mathrm{P}}*{V}_{\mathrm{E}}$$

Since GLuc exhibits a linear response over a wide range of enzyme concentrations (Harinen et al. 2013; Gordon et al. 2008; Golla et al. 2019; Meyer et al. 2021), a correlation factor ${F}_{\mathrm{C}}$ was introduced to relate the number of enzymes present in the polymersome experiment ${n}_{\mathrm{GLuc},\mathrm{P}}$ to the amount used during calibration ${n}_{\mathrm{GLuc},\mathrm{C}}$. This factor is defined as ${F}_{\mathrm{C}}=\frac{{n}_{\mathrm{GLuc},\mathrm{C}}}{{n}_{\mathrm{GLuc},\mathrm{P}}}$.

A fundamental prerequisite for investigating porin-mediated mass transport is that the enzymatic reaction within the polymersomes does not constitute the rate-limiting step. Therefore, Eq. 6 must be met, which indicates that the average number of GLuc enzymes per polymersome ${\overline{N} }_{\mathrm{GLuc},\mathrm{P}}$ multiplied by the turnover number ${k}_{\mathrm{cat}}$ must be smaller than the total transport rate per porin trimer ${k}_{\mathrm{transport}}$:

6 $${\overline{N} }_{\mathrm{GLuc},\mathrm{P}}* {k}_{\mathrm{cat}} < {k}_{\mathrm{transport}}$$

The resulting assay sensitivity indicated by the maximal mass transport calculated from Eq. 6 is summarized in Table 3. The turnover number ${k}_{\mathrm{cat}}$ of 17 s^−1^ was obtained from Borum et al. (2025).

Since the active enzyme concentration progressively decreases over the course of the assay due to inactivation, manifested as a decay in the observed luminescence signal, it is advisable to evaluate the luminescence evolution at the initial stage of the reaction. This approach is justified by the fact that the irreversible inactivation of the enzyme has been shown to correlate with the number of catalytic turnover cycles it performs (Dijkema et al. 2024, 2021). Consequently, we evaluated the initial increase in luminescence intensity ${v}_{\mathrm{GLuc}}$ for the different polymersomes – PolGLOmpF, PolGLOmpF∆, and PolGLPhoE.

In addition, although intra-vesicle diffusion could in principle contribute to systematic deviations in kinetic analysis, the intra-vesicular volume of the polymersomes is very small, implying that diffusion of small substrates and products occurs on a sub-millisecond timescale under the experimental conditions used. Therefore, diffusion within the vesicle interior is not expected to be rate-limiting under the experimental conditions used.

Upon determination of the mean initial slopes, the mean value obtained for the negative control PolGLControl was subtracted. The determination of the slopes is described in detail in the supplementary information. The resulting slope difference was then used to calculate the substrate concentration attributable specifically to transport through the porins by determining the corresponding substrate concentration $\normalsize {\mathrm{c}}_{\mathrm{S}}$ through rearrangement of Eq. 4.

To enable comparison of the luminescence signals from CLZ derivatives with the calibration curve established for native CLZ, we applied correction factors to account for substrate-dependent differences in the intrinsic bioluminescence quantum yield. Comparative studies indicate that chemical substitutions on CLZ primarily modulate the quantum yield, whereas the steady-state kinetic parameters remain within the same order of magnitude (Jiang et al. 2017; Loening et al. 2006). For *Renilla* luciferase, the C-6 4-amino-3-fluorophenyl derivative “B2” produces ~100-fold higher emission than DeepBlueC™ under equal conditions, yet the Michaelis-Menten constants are similar (B2, ${K}_{\mathrm{m}}=0.9 \pm 0.2$ µM; DeepBlueC™, ${K}_{\mathrm{m}}=0.6 \pm 0.1$ µM) (Jiang et al. 2017). Specifically, the measured intensities were multiplied by factors of 13.9 for CLZ-n and 11.7 for CLZ-I corresponding to their relative luminescence intensity of 7.2% ± 3.9% and 8.5% ± 0.86%, respectively, compared to native CLZ. These adjustments ensured accurate quantification across all substrates.

Since the transport rate ${k}_{\mathrm{transport}}$ multiplied by the concentration of porins ${c}_{\mathrm{Omp},\mathrm{P}}$ reconstituted into the polymersomes must equal the conversion rate of the enzyme, we can assume:

7 $${k}_{\mathrm{transport}}*{c}_{\mathrm{Omp},\mathrm{P}}= {v}_{\mathrm{GLuc}}={k}_{\mathrm{cat}}*{c}_{\mathrm{GLuc},\mathrm{i}}*\frac{{{\mathrm{c}}_{\mathrm{S}}}^{h}}{({{K}_{0.5}}^{h}+{{\mathrm{c}}_{\mathrm{S}}}^{h})}$$

This results in:

8 $${k}_{\mathrm{transport}}=\left({k}_{\mathrm{cat}}*{c}_{\mathrm{GLuc},\mathrm{i}}*\frac{{{\mathrm{c}}_{\mathrm{S}}}^{h}}{({{K}_{0.5}}^{h}+{{\mathrm{c}}_{\mathrm{S}}}^{h})}\right)*{{c}_{\mathrm{Omp},\mathrm{P}}}^{-1}$$

The concentration of porins relative to the total inner volume ${c}_{\mathrm{Omp},\mathrm{P}}$ is determined by:

9 $${c}_{\mathrm{Omp},\mathrm{P}}=\frac{{n}_{\mathrm{Omp}}}{{V}_{\mathrm{total},\mathrm{i}}}$$

Using the presented model, transport rates for all three porins were determined for the substrates CLZ, CLZ-I, and CLZ-n. The resulting values are presented with two significant digits in Table 4, providing a comparative overview of the channel-specific transport rates across the different substrates. The relative error of the mass transport rate for CLZ was calculated, including the error from densitometry and concentration measurement, while for the derivatives, the error resulting from the scaling factor was included as well. For the error values of the derivatives relative to CLZ, the relative errors determined for the mass transport of CLZ and the derivative were added.

**Table 4:** Summary of the determined mass transport rates per trimer of the reconstituted porins OmpF, OmpF∆, and PhoE for the native CLZ, as well as for the derivatives CLZ-n and CLZ-I. The transport rates for the derivatives relative to native CLZ are listed additionally

Reconstituted porin	CLZ native (± SEM), s^−1^	CLZ-n (± SEM), s^−1^	CLZ-n/CLZ native, %	CLZ-I(± SEM), s^−1^	CLZ-I/CLZ native, %
OmpF	10.8 ± 2.9	3.3 ± 1.9	0.31 ± 0.19	5.0 ± 0.73	0.46 ± 0.14
OmpF∆	78.0 ± 40.0	38.8 ± 23.2	0.50 ± 0.40	34.8 ± 6.2	0.45 ± 0.24
PhoE	2.8 ± 0.91	1.9 ± 1.1	0.68 ± 0.45	2.2 ± 0.43	0.79 ± 0.30

The determined mass transport rates for all the CLZ derivatives fall within the assay sensitivity determined by Eq. 6 and summarized in Table 3. Overall, the derivatives CLZ-n and CLZ-I exhibit a lower mass transport across all the porins relative to the native substrate. For wildtype OmpF, mass transport is reduced by more than half for both CLZ-n and CLZ-I. The values obtained for the derivatives relative to native CLZ range between 0.31 ± 0.19% and 0.68 ± 0.45% for CLZ-n and between 0.45 ± 0.24% and 0.79 ± 0.30% for CLZ-I, revealing less selectivity for PhoE compared to OmpF and OmpF∆ towards the two derivatives. The mass transport of OmpF∆ was at least 7 times higher relative to its wildtype porin OmpF for all CLZ derivatives, reaching 78.0 ± 40.0 s^−1^ for native CLZ. This is consistent with the observed luminescence signal in Fig. 3B relative to the inserted number of the porins per polymersome. For PhoE, the mass transport rates are about one-fourth of those for OmpF, consistent with the observed luminescence signals and the quantified porin insertion. The deletion variant shows at least 15 times increased mass transport across the derivatives relative to PhoE.

## Discussion

In this study, a novel assay for analyzing mass transport through porins reconstituted into synthetic membranes was established. The major advantage of this approach compared to whole cell measurements is the possibility to directly compare the transport properties of different porin variants without the distorting influence of the cellular background or different expression levels, which are difficult to resolve analytically, especially in the case of weakly expressed membrane proteins. To validate the approach, we compared three porins: the well-characterized benchmark OmpF, its deletion variant OmpF∆, and PhoE.

Recombinant expression of the porins was carried out under standardized conditions. Recombinant expression of OmpF in *E. coli* Omp8 via inclusion bodies resulted in 10–20 mg per liter cell culture (Miedema et al. 2004) reported yield, while the same study shows a reduced yield of 5–6 mg per liter cell culture for a mutant OmpF protein. This trend of porin mutants resulting in lower expression levels was equally observed in this study, while the yields are lower.

The embedding of porins in the membranes of artificial vesicles according to a bottom-up synthetic biology approach can enable a targeted investigation of their mass transport characteristics without having to resort to other physical phenomena such as conductivity or swelling behavior as proxies.

When designing such an assay with a targeted number of porins per vesicle, the protein-to-polymer ratio is a crucial factor. However, it is important to note that the efficiency of porin reconstitution varies strongly depending on the type of porin being used (Schwarzer et al. 2018). Additionally, factors such as the choice of polymer, membrane asymmetry, the use of additives, or hybrid approaches that combine lipids and polymers can significantly affect the efficiency of porin insertion (Wang and Tonggu 2015). Furthermore, although the orientation of reconstituted porins in the polymeric membrane is random in this assay, the large number of polymersomes ensures that orientation effects average out for weakly charged or neutral solutes, such as CLZ. The porin OmpF serves as a main model protein for understanding protein-membrane interactions in synthetic vesicles (Grzelakowski et al. 2009; Nardin et al. 2001; Klermund and Castiglione 2018). For this reason, there are various reports in the literature on the number of successfully reconstituted OmpF porins in polymersomes and liposomes. Edlinger et al. determined 11 OmpF porin molecules per vesicle for a 125 nm diameter polymersome with the polymer PMOXA_6_-*b*-PDMS_44_-*b*-PMOXA_6_ (Edlinger et al. 2017). In the same range, Nardin et al*.* reported 5 to 20 OmpF channels per 250 nm polymersome (Nardin et al. 2001). The reconstitution of OmpF porins into liposomes (diameter < 100 nm) resulted in 0.4 ± 0.03 OmpF trimers per liposome (Zhang et al. 2025). To investigate mass transport across compartment boundaries, it is essential that the mass transport and not the detection reaction is the rate-determining step. Therefore, we kept the number of porins per vesicle in a very low range in this study, which in the case of OmpF was 0.20 ± 0.021 trimers per polymersome.

A direct comparison of the insertion efficiencies of different porins was conducted by Schwarzer et al. (2018), resulting in a 1.4 times higher insertion efficiency of the PhoE porin compared to OmpF. In total, 44 PhoE molecules per polymersomes could be reconstituted. In this work, PhoE exhibited over threefold higher insertion into the polymeric membrane, relative to OmpF. This difference could stem from different reconstitution methods being used. While Schwarzer et al. reconstituted the polymersomes post-production, in this work, the insertion takes place during polymersome formation (Schwarzer et al. 2018). The reconstitution of the variant OmpF∆ into the polymeric membrane is reduced by a factor of almost seven. It is known that mutants of membrane porins may impair membrane integration in both native and artificial contexts compared to their wildtype counterparts. A previous study of the deletion variant OmpF∆ also showed a lower efficiency of insertion compared to the wildtype OmpF, although the extent of this was not quantified (Saint et al. 1996). However, the insertion of the mutant OmpF G119D by Klermund et al. (2017) resulted in a 7 times lower insertion compared to OmpF (Vrouenraets et al. 2006), similar to the relative insertion of the mutant in this study.

In line with literature, the observed luminescence data was fitted to the Hill equation. A Hill coefficient of 1.8 ± 0.2 was reported (Larionova et al. 2018), which is not fundamentally different from the value of 1.4 (95% CI [0.83, 2.1]) observed in this study. The observed ${k}_{0.5}$ value of 9.8 µM (95% CI [6.2 µM, 42.9 µM] is also in the same range as the literature, where a value of 12.6 ± 2.8 µM was reported (Inouye 2018). Next, we applied this framework to evaluate substrate transport across the polymeric membrane. Overall, the uptake of single OmpF varies between one and a few hundred molecules per second per channel for antibiotics (Winterhalter 2021). For the slightly cation-selective porin OmpF, the determined mass transport of 10.8 ± 2.7 s^−1^ trimer^−1^ represents a realistic value, consistent with previously reported transport rates for comparable small molecules, such as fluoroquinolone antibiotics, particularly ciprofloxacin, norfloxacin, and enrofloxacin, which have comparable molecular mass of 331.3 Da, 319.3 Da, and 359.4 Da, respectively, a largely neutral charge at physiological pH, and a high aromatic content. Reported trimeric OmpF-mediated transport rates for these molecules range from 7 ± 0.8 to 10 ± 1 molecules s^−1^ for reconstructed porins in lipid membranes (Mahendran et al. 2010; Cama et al. 2015), supporting the notion that the rate observed in our system is well within the expected range for passive diffusion through this porin. The deletion mutant OmpF∆ has previously been investigated using liposome-swelling assays with various sugars, where it exhibited a 3—12-fold increase in permeation compared to wild-type OmpF (Saint et al. 1996), which is consistent with the enhancement observed in our study. Comparable deletion variants have been analyzed in whole-cell assays using various antibiotics larger than 600 Da and demonstrated significantly enhanced permeability, relative to wild-type OmpF (Benson et al. 1988). For native CLZ, which is an uncharged, highly aromatic molecule with multiple heterocycles, the diffusion does not benefit from the attractive interactions that arise between the zwitterionic molecules and charged residues at loop L3 (Eppens et al. 1997; Pagès et al. 2008; Vergalli et al. 2020). Consequently, the higher mass transport of the deletion variant should only be attributed to the lower sterical hindrance in the pore eyelet region and not to the altered electrical field. Regarding the porin PhoE, the observed mass transport rate was almost half that of OmpF. Although direct comparative data between these two porins is limited, this difference can be reasonably attributed to their distinct selectivity profiles. OmpF, while mildly cation-selective, functions as a general diffusion channel and is relatively permissive to neutral molecules. In contrast, PhoE exhibits moderate anion selectivity and is primarily adapted for the transport of negatively charged solutes such as phosphate and dicarboxylates (Nikaido 2003; Pagès et al. 2008). Consequently, the slightly reduced permeability of PhoE for neutral substrates such as CLZ is consistent with its known transport preferences.

The overall decrease in mass transport observed for the derivatives CLZ-n and CLZ-I relative to the native CLZ can be rationalized by their altered physiochemical properties. Both derivatives possess higher log P values, indicating reduced polarity, and increased steric bulk compared to native CLZ. These factors likely affect not only the partitioning of the molecules into the membrane or the pore environment but also their orientation and interaction with the electrostatic field within the eyelet region, resulting in reduced transport efficiency (Bajaj et al. 2017).

## Conclusions

Porins act as key gateways for nutrient uptake and antibiotic influx in Gram-negative bacteria and play a fundamental role in determining outer membrane permeability. Nevertheless, quantitative analysis of transmembrane transport remains inherently challenging due to the complexity and variability of cellular systems. In this context, synthetic bottom-up approaches offer a powerful alternative to traditional whole-cell assays, minimizing biological complexity and eliminating confounding influences from native cellular processes. Embedding porins into synthetic vesicle membranes allows the direct study of their mass transport and is a robust alternative to whole-cell experiments, without relying on indirect proxies such as conductivity or swelling. By encapsulating GLuc within the polymersomes, the translocation of the substrate CLZ across the polymeric membrane, both in the absence and presence of reconstituted porins, could be directly and quantitatively monitored. The deletion variant OmpF∆ showed more than seven times higher mass transport relative to OmpF across all derivatives investigated. Furthermore, an absolute mass transport of 79 molecules s^−1^ for this porin could be determined for the first time.

To further expand the applicability of this assay, one possibility is to extend the substrate range to charged molecules, enabling a more detailed analysis of porin selectivity arising from mutations, such as in the OmpF∆ variant. While this study already addressed substrates of slight and notable differences in size and hydrophobicity, respectively, the broad substrate scope of luciferases offers the opportunity to test an even wider chemical range, including compounds of different charge states and functional groups, that mimic antibiotics. Such diversification would allow a more comprehensive mapping of porin transport properties, facilitate the characterization of clinically relevant mutants, and open new paths for exploring membrane permeability in both fundamental and applied nanobiotechnological contexts and pharmacologically relevant studies (Pagès et al. 2008).

## Supplementary information

Below is the link to the electronic supplementary material.

ESM 1 (DOCX 4.42 MB)

## Data Availability

The raw data supporting the conclusions of this article is available in the Zenodo repository 10.5281/zenodo.17047081.

## Abbreviations

AA: Amino acid
a.u.: Arbitrary units
CLZ: Coelenterazine
DLS: Dynamic light scattering
EDTA: Ethylendiamintetraacetic acid
GLuc: *Gaussia* Luciferase
IMAC: Immobilized metal affinity chromatography
IPTG: Isopropyl‐β‐D‐thiogalactopyranoside
LB: Lysogeny broth
MW: Molecular weight
LDAO: N,N-dimethyldodecylamine N-oxide
O-POE: N-Octyl-oligo-oxyethylene
OD_600_: Optical density at 600 nm
Omp: Outer membrane protein
OmpF: Outer membrane protein F
PMSF: Phenylmethylsulfonyl fluoride
PBS: Phosphate buffered saline
PhoE: Phosphoporin E
PDI: Poly dispersity index
PC: Polycarbonate
SEC: Size-exclusion chromatography
SEM: Standard error of the mean
SDS-PAGE: Sulfate–polyacrylamide gel electrophoresis
