# Supplementary material for: Quantitative assessment of porin-mediated solute transport in biomimetic membranes
Source: Appl Microbiol Biotechnol. 2025 Dec 19;109(1):278. doi: 10.1007/s00253-025-13666-0 (PMC12718255; doi:10.1007/s00253-025-13666-0)
Supplement: Supplementary file 1 — (DOCX 4.42 MB) [file 253_2025_13666_MOESM1_ESM.docx]

Supporting Information

**Quantitative assessment of porin-mediated solute transport in biomimetic membranes**

Maike Scherer, Teena tom Dieck, Leila Pourtalebi, Robert Schober, Maximilian Schäfer, and Kathrin Castiglione*

Corresponding Author

* Prof. Kathrin Castiglione

Address: Institute of Bioprocess Engineering, Friedrich-Alexander-Universität Erlangen-Nürnberg, Paul-Gordan-Straße 3, 91052 Erlangen, Germany

E-mail: [kathrin.castiglione@fau.de](mailto:kathrin.castiglione@fau.de)

**Contents**

[1 Results of the purification of the porins and of GLuc 3](#_Toc207626663)

[1.1 Purification of the porin OmpF 3](#_Toc207626664)

[1.2 Purification of the deletion variant OmpF∆ 3](#_Toc207626665)

[1.3 Purification of the porin PhoE 4](#_Toc207626666)

[1.4 Purification of GLuc 5](#_Toc207626667)

[2 Additional results from the polymersome analysis 6](#_Toc207626668)

[2.1 Densitometric analysis of the porin insertion 6](#_Toc207626669)

[2.1.1 PolGLControl 6](#_Toc207626670)

[2.1.2 PolGLOmpF 7](#_Toc207626671)

[2.1.3 PolGLOmpF∆ 8](#_Toc207626672)

[2.1.4 PolGLPhoE 8](#_Toc207626673)

[3 Mass transport 9](#_Toc207626674)

[3.1 Determination of slope for derivatives measurements and PolGLControl with native CLZ 9](#_Toc207626675)

[3.2 Determination of the initial slope for porin containing samples with native CLZ 10](#_Toc207626676)

[3.3 Data of free GLuc for the calibration curve 10](#_Toc207626677)

[3.4 Results of the luminescence assay to determine mass transport of the porins 10](#_Toc207626678)

[4 References 11](#_Toc207626679)

# Results of the purification of the porins and of GLuc

The PAGE gels of the purification of the porins OmpF, OmpF∆, and PhoE as well as the enzyme GLuc are included in this section. The band corresponding to the porins or the enzyme were quantified. The calculated molar mass for the proteins was determined using ProtParam.

## Purification of the porin OmpF

Figure S1 includes the PAGE analysis of the purification of OmpF. The porin is visible around 38kDa, indicated by a blue arrow, which corresponds to its molecular weight of 38.8 kDa. OmpF appears as a double band due to incomplete denaturation [1].


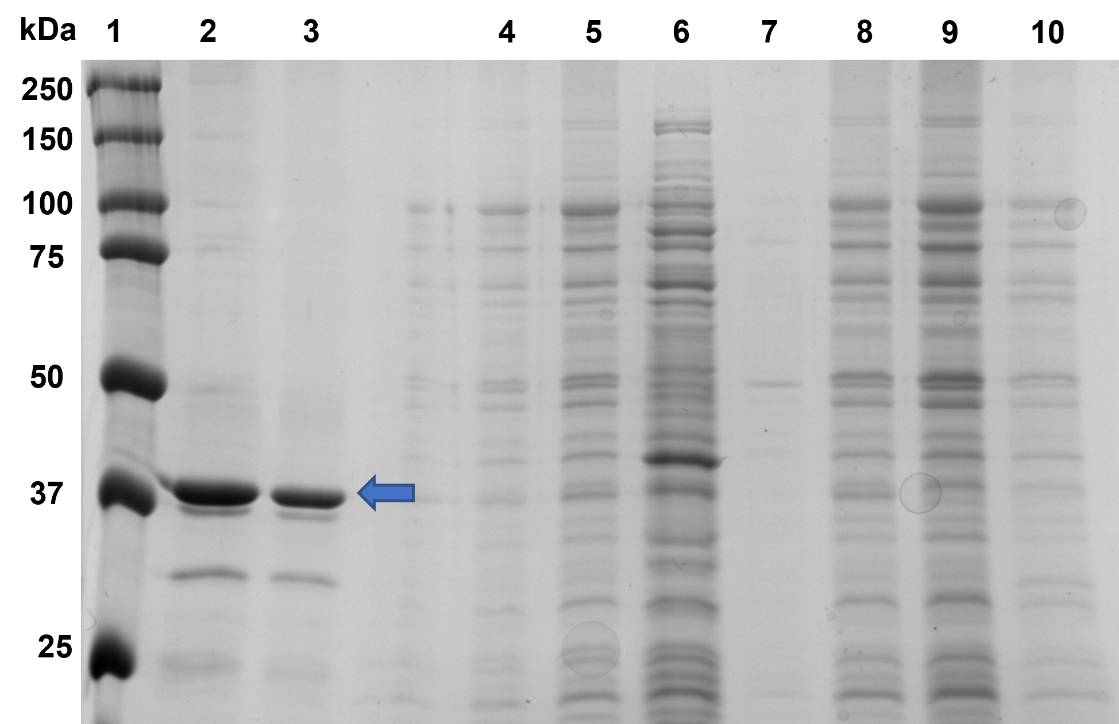


Figure S1 - PAGE gel of the purification of OmpF. The lanes depict the applied marker (1), the purified OmpF porin in a 1:5 and a 1:10 dilution in (2) and (3), respectively, the pellet in a 1:50 (4) and a 1:25 dilution (5), the supernatant (6) of the 1^st^ ultracentrifugation step, the pellet (7) and supernatant (8) of the 2^nd^ ultracentrifugation step, a sample of the filtrate application during the IMAC 1:10 diluted (9), a sample of the wash step 1:10 diluted (10). The lanes (6), (7), and (8) were applied in a 1:25 dilution.

## Purification of the deletion variant OmpF∆

Figure S2 shows the PAGE analysis of the purification of the deletion variant OmpF∆. The porin is visible around 38kDa which, indicated with a blue arrow, corresponds to its molecular weight of 38.2 kDa.


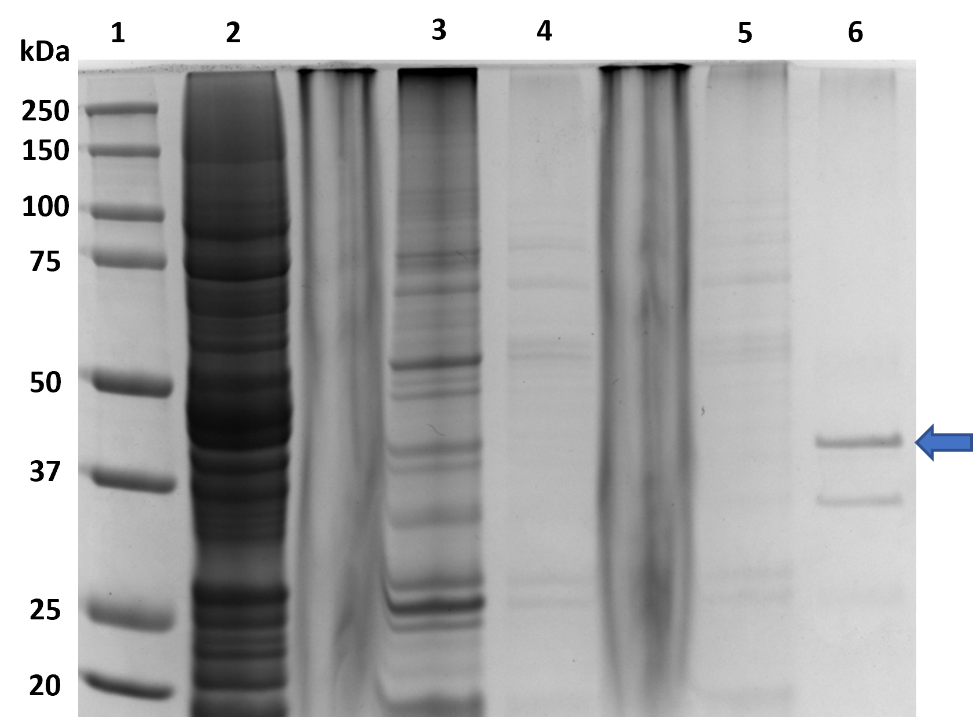


Figure S2 - PAGE gel of the purification of OmpF∆. The lanes depict the applied marker (1), supernatant of the 1^st^ ultracentrifugation step (2), the pellet of the 2^nd^ ultracentrifugation step (3), the filtrate that was applied to the IMAC 1:25 diluted (4), sample of the wash step during the IMAC 1:10 diluted (5), and the purified OmpF∆ porin (6). (2) and (3) were diluted 1:10.

## Purification of the porin PhoE

Figure S3 includes the PAGE analysis of the purification of PhoE. The porin is visible around 38kDa, indicated by a blue arrow, which corresponds to its molecular weight of 38.6 kDa. PhoE appears as a double band due to incomplete denaturation [1].


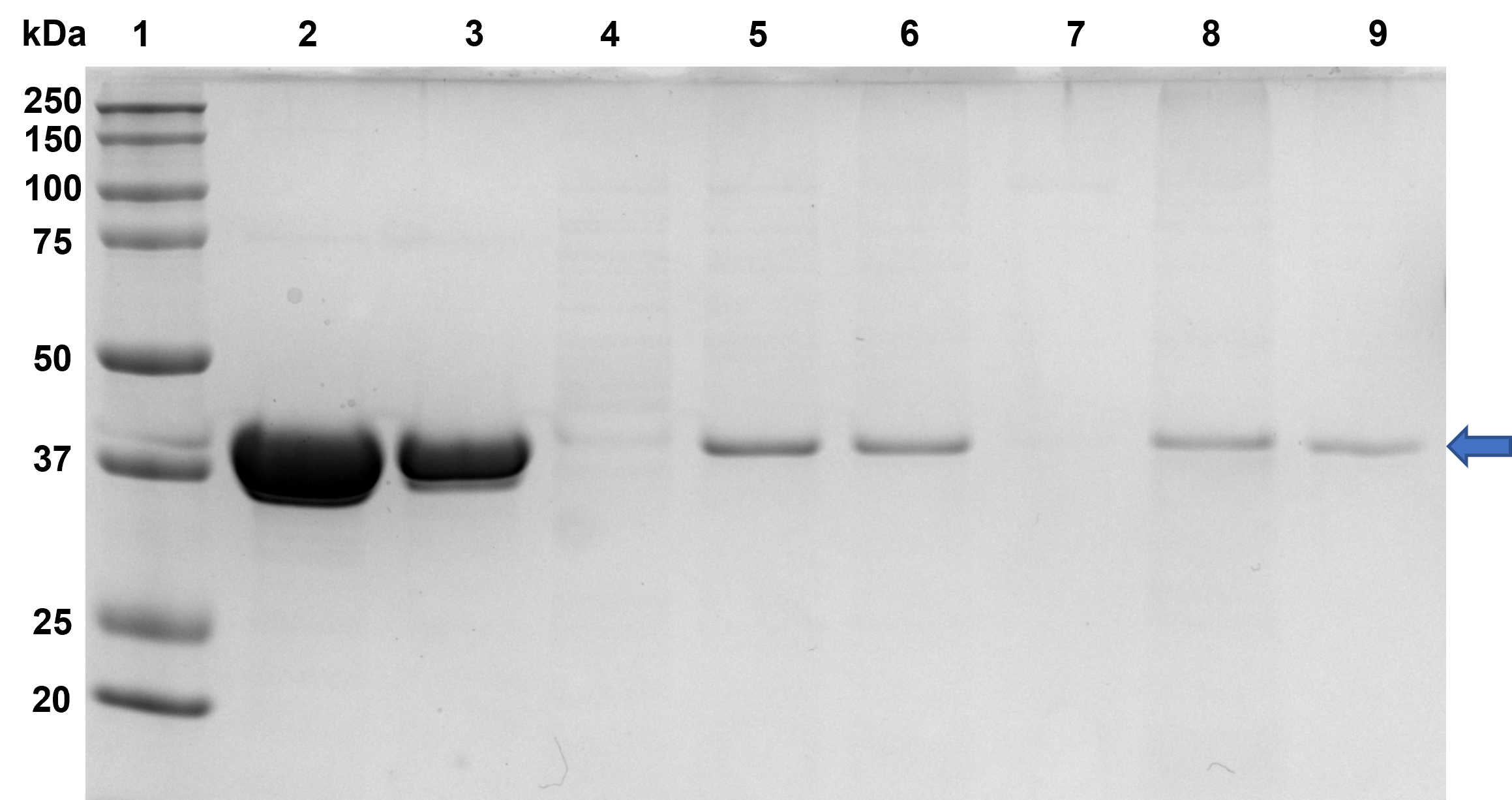


Figure S3 - PAGE gel of the purification of PhoE. The lanes depict the applied marker (1), the purified PhoE porin in a 1:5 and a 1:10 dilution in (2) and (3), respectively, the supernatant (4) and the pellet (5) of the 1^st^ ultracentrifugation step, the supernatant (6) and pellet (7) of the 2^nd^ ultracentrifugation step, the filtrate that was applied to the IMAC 1:10 diluted (8), a sample of the wash step 1:10 diluted (9). (4), (5), (6), and (7) were diluted 1:25.

## Purification of GLuc

Figure S4 shows the PAGE analysis of the purification of GLuc. Gluc can be observed right below the 20 kDa marker band, which corresponds to its molecular mass of 19.0 kDa.


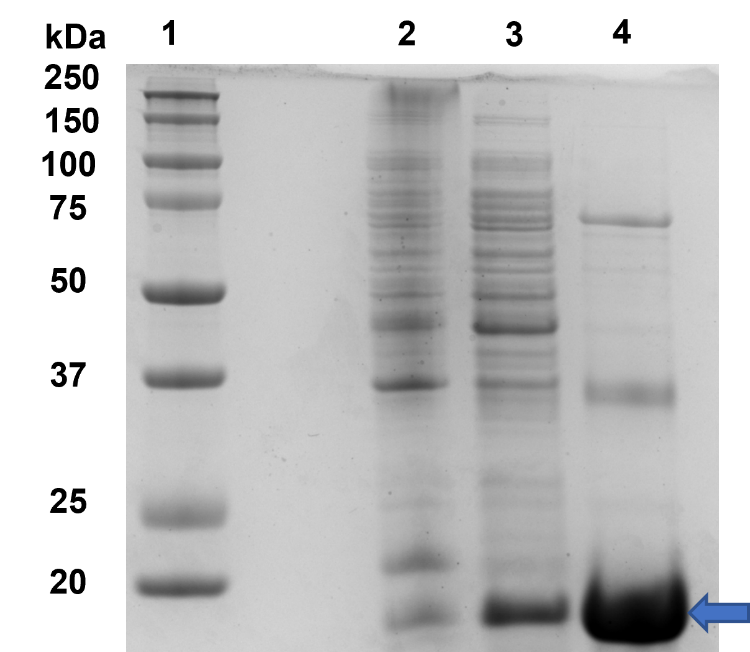


Figure S4 - PAGE gel of the purification of GLuc. The lanes depict the applied marker (1), a sample from the pellet (2), the applied filtrate (3) and the purified GLuc (4). Samples in (2) and (3) were diluted 1:20.

# Additional results from the polymersome analysis

Figure S5 shows the results of the DLS measurements to determine the quality and the diameter of the polymersome solution.


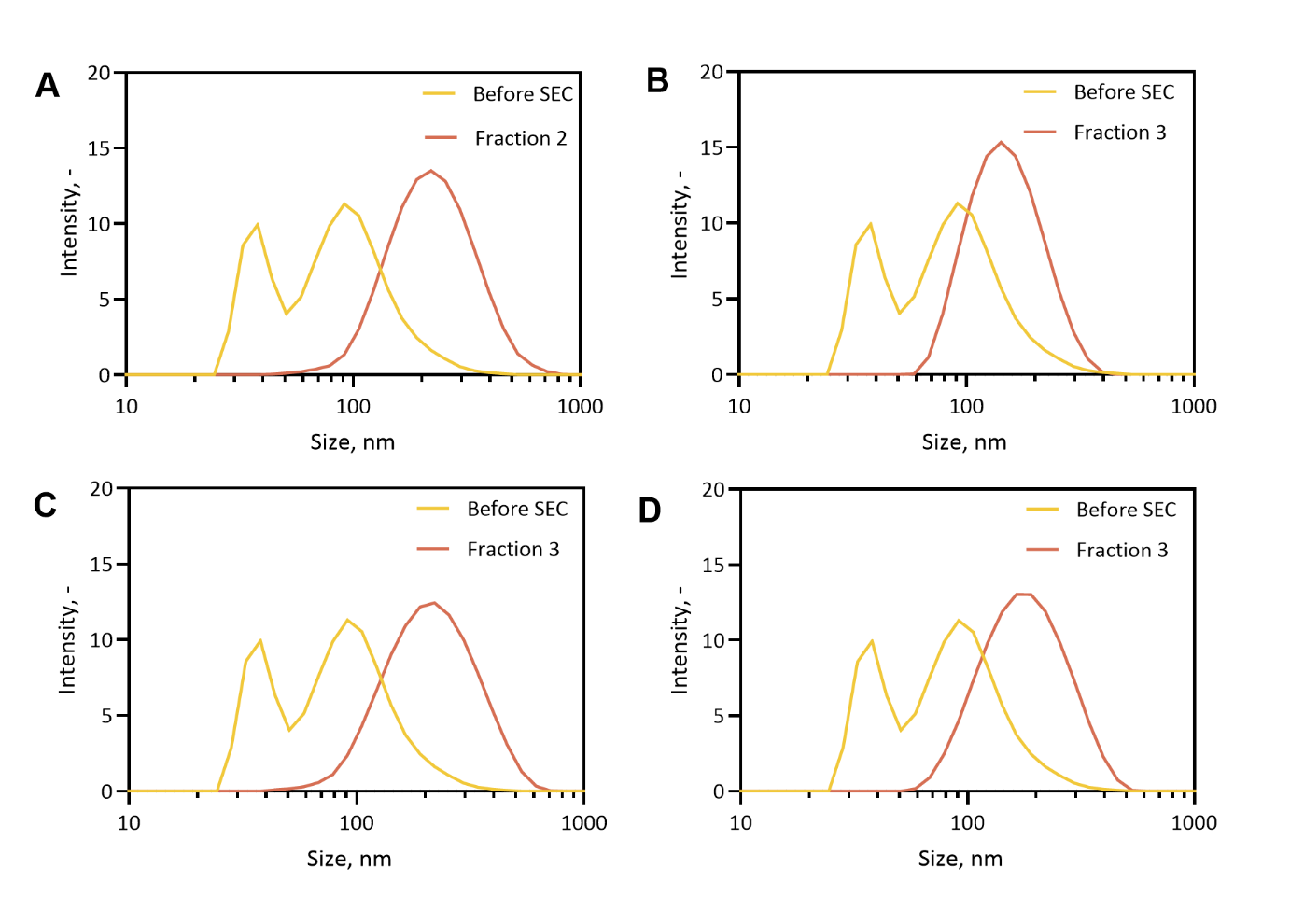


Figure S5 - Results from DLS-measurements for the polymersomes PolGLControl (**A**), PolGLOmpF (**B**), PolGLOmpF∆ (**C**), and PolGLPhoE (**D**). The results of the size distribution before SEC, after two rounds of extrusion are indicated in yellow, depicting two maxima, one for the smaller micelles around 30 nm and one for polymersomes. Only the fraction of the SEC that has been used for the experiments is shown. The majority of the micelles is depleted in the purified fractions.

## Densitometric analysis of the porin insertion

The PAGE gels of the densitometric analysis of the polymersomes PolGLControl, PolGLOmpF, PolGLOmpF∆, and PolGLPhoE are shown in this section.

### PolGLControl

Figure S6 contains the PAGE analysis of the control polymersomes PolGLControl with no inserted porins on a LDS PAGE gel with coomassie staining (A) and an LDS PAGE with SYPRO ruby staining (B). Gluc is visible in B below the 25 kDa marker band.


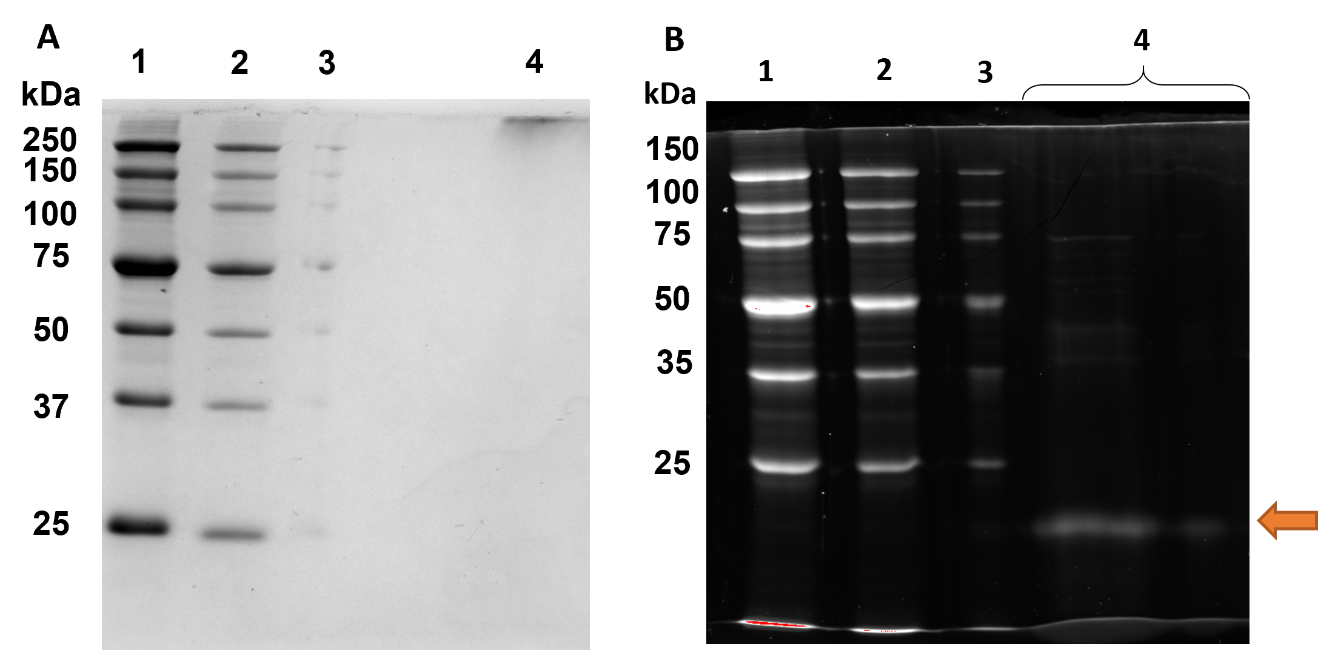


Figure S6- PAGE analysis of the control polymersomes with no inserted porins on a LDS PAGE gel with coomassie staining (A) and an LDS PAGE with SYPRO ruby staining (B). From the marker 1 µg, 0.5 µg, and 0.1 µg were loaded into lanes 1, 2 and 3, respectively. The PolGLControl sample was applied to lane 4. Gluc is visible below the 25 kDa band (B).

### PolGLOmpF

Figure S7 shows the PAGE analysis of the polymersomes PolGLOmpF. OmpF is visible around 38 kDa, indicated by a blue arrow, confirming successful insertion of the porin into the polymeric membrane. OmpF appears as a double band due to incomplete denaturation [1].


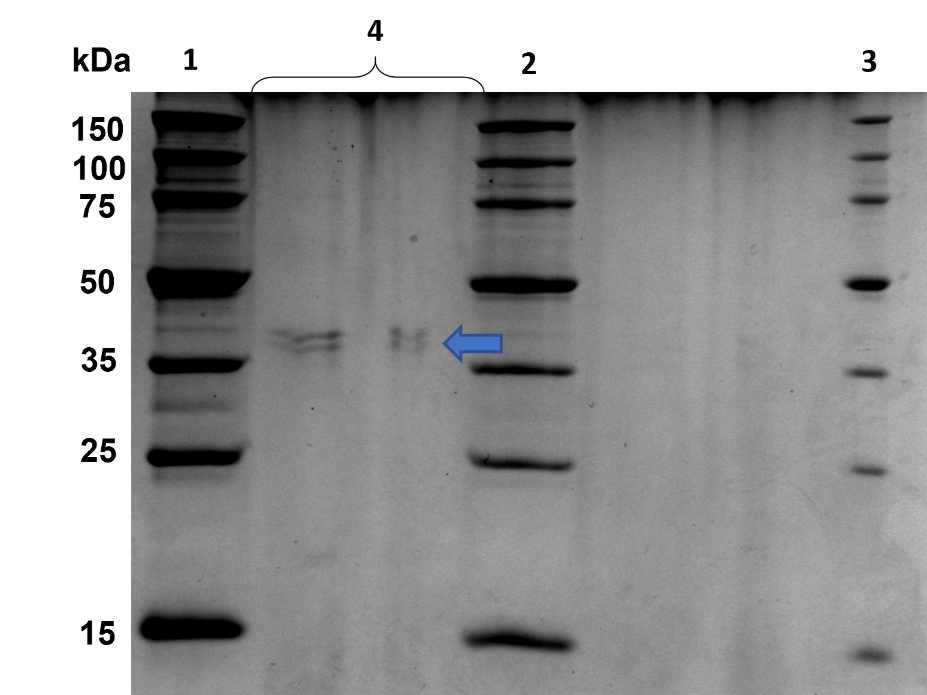


Figure S7 - PAGE analysis of the insertion of OmpF into the polymeric membrane. From the marker 1 µg, 0.5 µg, and 0.1 µg were loaded into lanes 1, 2, and 3, respectively. The PolGLOmpF sample was applied to two lanes, which are collectively referred to here as lane 4.

### PolGLOmpF∆

Figure S8 shows the PAGE analysis from the polymersomes PolGLOmpF∆. The image has been inverted so that the bands around 38 kDa for OmpF∆ are more visible, indicated by a blue arrow. This confirms successful insertion of the porin into the polymeric membrane. The porin appears as a double band due to incomplete denaturation [1].


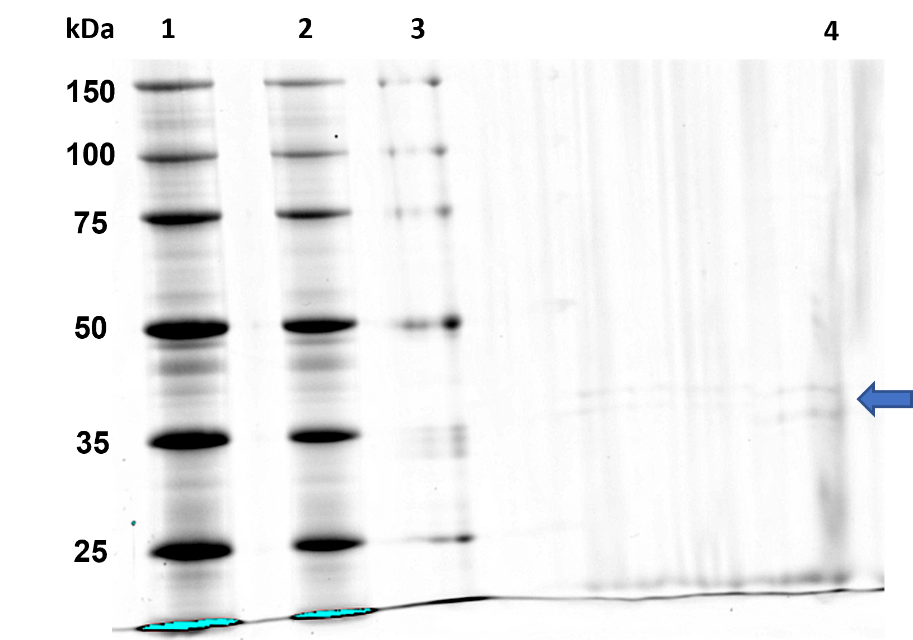


Figure S8 - PAGE analysis of the insertion of OmpF∆ into the polymeric membrane. From the marker 1 µg, 0.5 µg, and 0.1 µg were loaded into lanes 1, 2, and 3, respectively. The PolGLOmpF∆ sample was applied to lane 4.

### PolGLPhoE

Figure S9 showes the PAGE analysis of the polymersomes PolGLPhoE. PhoE is visible around 38 kDa, indicated by a blue arrow, confirming successful insertion of the porin into the polymeric membrane. Again, below the 25 kDa band of the marker, a faint band is visible, showing successful GLuc insertion.


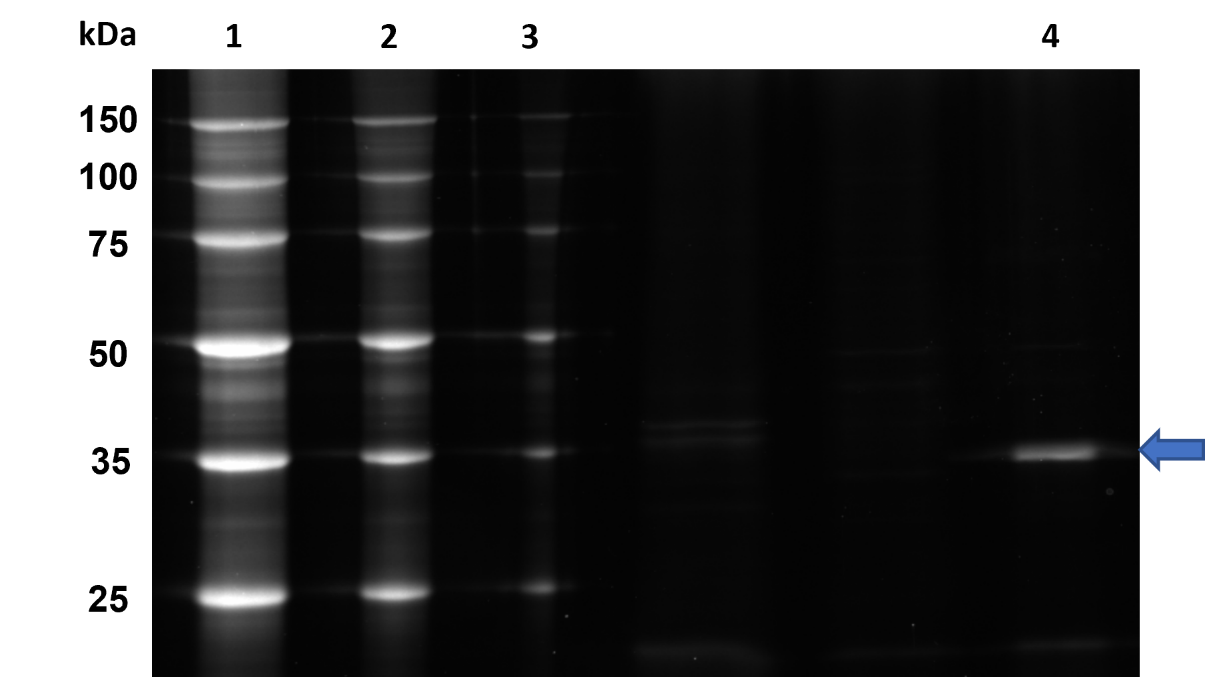


Figure S9 - PAGE analysis of the insertion of PhoE into the polymeric membrane. From the marker 1 µg, 0.5 µg, and 0.1 µg were loaded into lanes 1, 2, and 3, respectively. The PolGLPhoE sample was applied to lane 4.

# Mass transport

## Determination of slope for derivative measurements and PolGLControl with native CLZ

We applied the following procedure for the control polymersomes PolGLControl and the measurements of the porin containing polymersomes with the CLZ derivatives and CLZ-n. First, we smoothed the experimental curves using an averaging filter of tab length $w$ to reduce the effect of noise on the obtained slopes. Next, a simple linear curve was fitted on slices of length $b$ of the experimental data. This procedure was applied to each time step, i.e., with overlapping slices. The curve yielding the highest slope was then assumed to be the initial slope of the reaction. For the variables $w$ and $b$ we have chosen the following values shown in Table S1.

Table S1 - Variables w and b for the determination of initial slope for the substates CLZ and CLZ-n.

| Derivative | $w$ | $b$ |
| --- | --- | --- |
| CLZ | - | 15 |
| CLZ-n | 5 | 5 |

## Determination of the initial slope for porin containing samples with native CLZ

The samples PolGLOmpF, PolGLOmpF∆, and PolGLPhoE exhibited very high luminescence intensites with sharp initial increases. For native CLZ, the slope was determined using the initial increase with the lowest deviation.

## Data of free GLuc for the calibration curve

In Figure S10, the complete results of the measurements of the luminescence intensity in samples with free GLuc are shown.


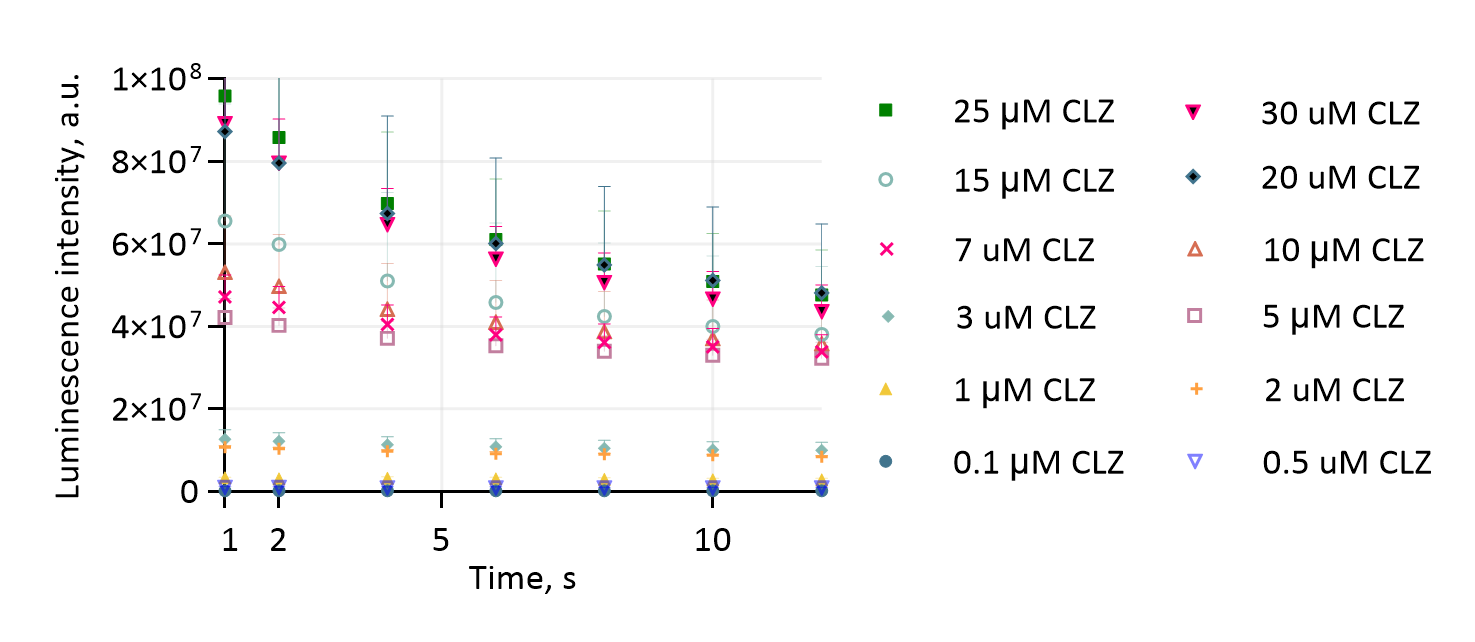


Figure S10 – Results of the luminescence measurements of free GLuc for the calibration curve and the extrapolated value at 1 s. The luminescence intensity was recorded for the CLZ-concentrations.

## Results of the luminescence assay to determine mass transport of the porins

Figure S11 A depicts the complete raw data of measured luminescence intensity signals for the calculation of the mass transport of the porins OmpF, OmpF∆, and PhoE, as well as the controls of PolGLControl and PBS. The two biological replicates of PhoE are displayed in Figure S11 B, while the maximal fluorescence intensity and concentration of the replicates is summarized in Table S2.

Table S2 – Comparison of the maximum luminescence intensity and concentration in polymersomes per mL of two biological replicates of PolGLPhoE.

|  | **Max. intensity, a.u.** | **Concentration, pol mL^-1^** |
| --- | --- | --- |
| Biol. replicate of PolGLPhoE 1 | 5.1*10^7^ | 3.7*10^12^ |
| Biol. replicate of PolGLPhoE 2 | 5.6*10^6^ | 3.7*10^11^ |


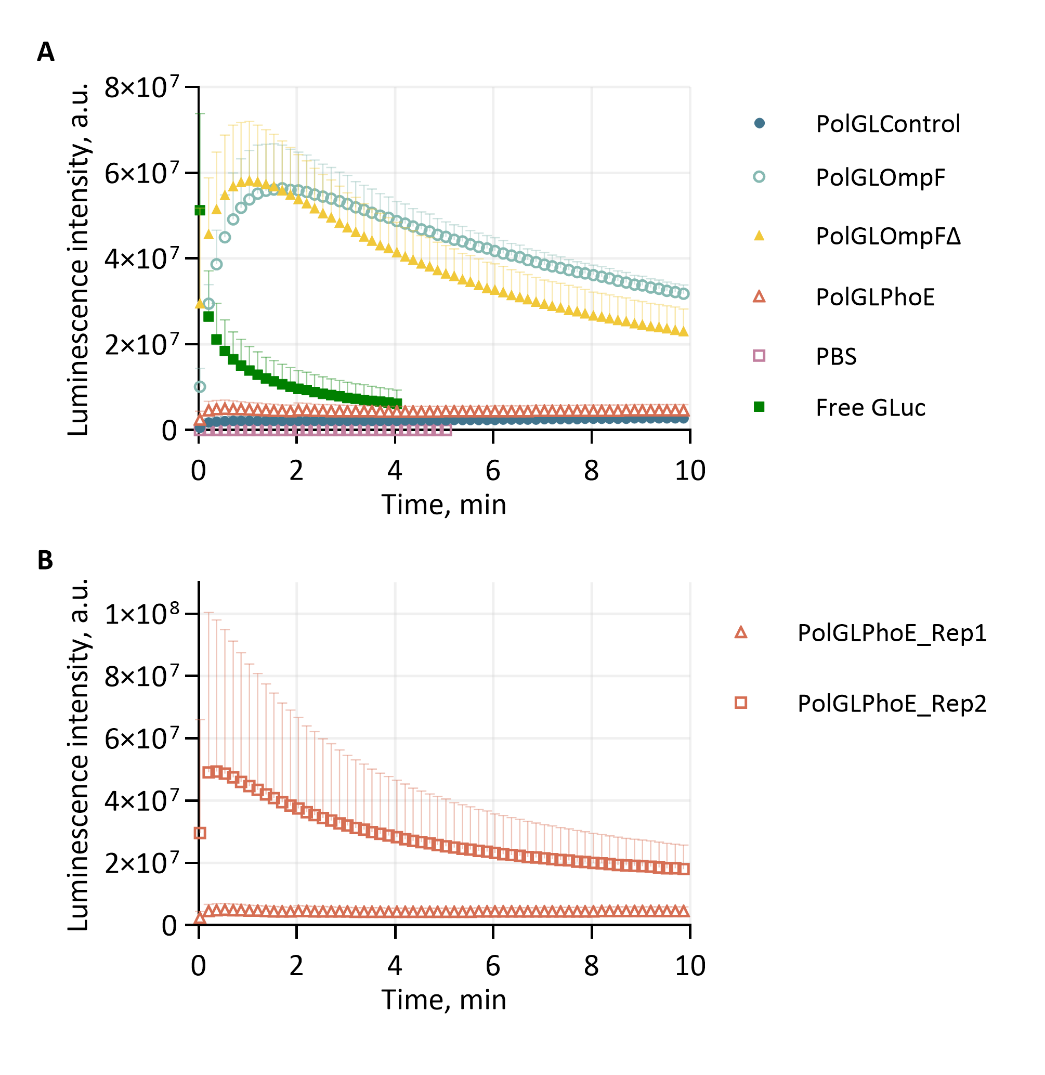


Figure S11 – Raw data of the measured luminescence intensity signals over time from the mass transport analysis. Only every fifth recorded data point is shown in **A** and **B**, to facilitate visualization. Additionally, standard deviations are displayed only above the data points. The luminescence intensity over time upon the injection of 80 µM CLZ (**A**) to a polymersome solution with reconstituted porins OmpF, OmpF∆, and PhoE resulted in the signal PolGLOmpF (turquoise circles), PolGLOmpF∆ (yellow filled triangles), and PolGLPhoE (red triangles) respectively. The flash-type luminescence signal of 0.05 µM free GLuc upon injection of 15 µM CLZ is shown with green filled rectangles. As a reference, the negative control of polymersomes with no porins PolGLControl (cyan filled circles) and the buffer control (violet rectangles) are added. In **B**, two biological replicates of PolGLPhoE are plotted to display the scaled difference in luminescence intensity upon varying polymersome concentration.

References

1. Rath A, Glibowicka M, Nadeau VG, Chen G, Deber CM. Detergent binding explains anomalous SDS-PAGE migration of membrane proteins. Proc Natl Acad Sci U S A. 2009;106:1760–5. doi:10.1073/pnas.0813167106.
